# Hierarchical Reconfigurable Metasurface Based on Scenario‐Guided Functional Modules and Programmable Core

**DOI:** 10.1002/advs.202524154

**Published:** 2026-03-13

**Authors:** Lihao Zhu, Jiaqi Han, Zhe Zheng, Qiang Feng, Dexiao Xia, Xiangjin Ma, Yajie Mu, Guoliang Luo, Rui Li, Haixia Liu, Hao Xue, Long Li

**Affiliations:** ^1^ Key Laboratory of High‐Speed Circuit Design and EMC School of Electronic Engineering Ministry of Education Xidian University Xi'an China

**Keywords:** hierarchical reconfigurable metasurface, multifunctional manipulation, scenario‐guided design, switchable scattering

## Abstract

The conventional metasurface remains functionally inflexible, as their platform are typically optimized and designed for specific scenarios, necessitating a complete redesign when operational parameters and function change—a process that incurs significant time and costs. To overcome the fundamental limitation, we propose a hierarchical reconfigurable metasurface architecture (HRMA) to achieve comprehensive electromagnetic parameter modulation and on‐demand polymorphic function switching. The reconfigurable metasurface is divided into a programmable core (PC) and scenario‐guided functional modules (FMs). The PC differs from traditional switches in terms of structure, performance, and manner of use, exhibiting significant flexibility and reusability. The low‐cost FM is attached to the PC, protecting it and endowing the metasurface with specific functional requirements. Based on this architecture, we achieve polymorphic functionality, effectuate extreme bandwidth coverage, and verify the possibility of comprehensive electromagnetic parameter regulation through a single hardware platform. This architecture provides a solution to orchestrate polymorphic scattering in diverse scenarios, offering a scalable design paradigm for next‐generation reconfigurable microwave devices and systems.

## Graphical abstract

This paper proposes a hierarchical reconfigurable metasurface architecture (HRMA) to achieve comprehensive electromagnetic parameter modulation and on‐demand polymorphic function switching. The reconfigurable metasurface is divided into a programmable core (PC) and scenario‐guided functional modules (FMs), exhibiting significant flexibility and reusability. Based on this architecture, polymorphic functionality, extreme bandwidth coverage, possibility of comprehensive electromagnetic parameter regulation through a single hardware platform are achieved.

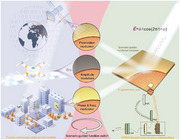

## 1 Introduction

Since the inception of digital metasurfaces [1], reconfigurable metasurfaces (RMS) have ushered in a paradigm shift for scattering and radiation control, revolutionizing the electromagnetic (EM) wave modulation in the spatio‐temporal domain with significant functional agility [2, 3, 4, 5, 6]. State reconfiguration pioneers dynamic adaptability in metasurfaces through external regulation, whereas encoding quantization establishes a bidirectional interface bridging physical implementation with digital control frameworks. On the physical level, RMS stimulated by various methods such as electronic control, light control, and thermal control have been proposed [7, 8, 9, 10, 11, 12, 13, 14]. Gradually, RMS has emerged as a versatile physical platform for multi‐domain EM control [15, 16, 17], spanning diverse application domains including RIS‐enhanced wireless communication systems, RCS reduction stealth devices, compressive sensing imaging platforms, and multi‐dimensional positioning networks [18, 19, 20, 21, 22, 23, 24, 25, 26, 27, 28, 29, 30, 31]. A multitude of applications have been progressively demonstrated through successive generations of RMS, showcasing an evolving landscape of EM control capabilities.

Nevertheless, as an EM modulation platform, functional metasurfaces have yet to fulfill their potential. Current design paradigms remain predominantly limited to shallow encoding‐state modulation with only marginal functionality adaptation capabilities. Although some metasurfaces with functional switching have been proposed, they are designed in predetermined scenarios and often cannot meet practical needs once there is a slight change in functional requirements [32, 33, 34, 35, 36]. Some learning algorithms have been used for rapid adaptation of metasurfaces across different scenarios [37, 38]. However, limited hardware functionality will first limit the use of the proposed algorithm, and the complex design and high‐cost processing requirements for corresponding hardware in different scenarios have not been addressed. The traditional design method focuses on target setting and parameter optimization around reconfigurable devices such as diodes, and a fixed angle field of view leads to insufficient flexibility. On one hand, the change in functional requirements requires the reconfigurable surface to be redesigned and reprocessed entirely, resulting in long time cycles in design and processing and high‐cost consumption caused by the tunable devices. On the other hand, the evolving requirements for EM design parameters also pose significant challenges to the multispectral applicability of metasurfaces. Key performance metrics—such as operational bandwidth and modulation depth—exhibit strong scenario dependence, invariably leading to identical design limitations [39, 40, 41]. According to the Bode‐Fano condition [42], for specific types of complex loads, passive networks cannot achieve perfect matching within infinite bandwidth. Scenario‐specific fluctuations in function and parameter requirements prevent unified solutions [43, 44], while current reconfigurable paradigms remain inadequate and inflexible for constructing intelligent scattering responses across dynamic scenarios.

In this article, we report a hierarchical reconfigurable metasurface architecture (HRMA), introducing modular electronics into scattering regulation and enabling cross‐scenario reconfigurable metasurface design with extremely low cost and fast processing cycles. A novel reconfigurable load, named programmable core (PC), is constructed to ensure operational portability across diverse application requirements. Polymorphic function and parameter requirements are implemented through low‐cost functional modules (FM) loading hierarchically with the PC, which also serves as a cover to protect it. Compared to the traditional architecture, HRMA demonstrates its superiority, as the operating parameters of the RMS (such as working frequency) and functions can be switched through a simple and low‐cost FM design. Through this paradigm, we demonstrate comprehensive regulation of EM parameters with one PC and five FMs. We present experimental verification of a bandwidth‐migratable phase modulator spanning the ultra‐wideband, a wideband amplitude modulator with a tunable perfect absorption peak, and a polarization converter for the entire polarization plane—all while maintaining the reusability of reconfigurable layers across function transitions. This work shows that the HRMA not only circumvents the performance‐complexity tradeoffs plaguing conventional designs but also establishes a transferable platform for realizing multifunctional scattering control positioned to redefine system integration paradigms in communications, detection, and adaptive stealth technologies.

## 2 Results

### 2.1 Principle of the Hierarchical Reconfigurable Metasurface Architecture

**FIGURE 1:**
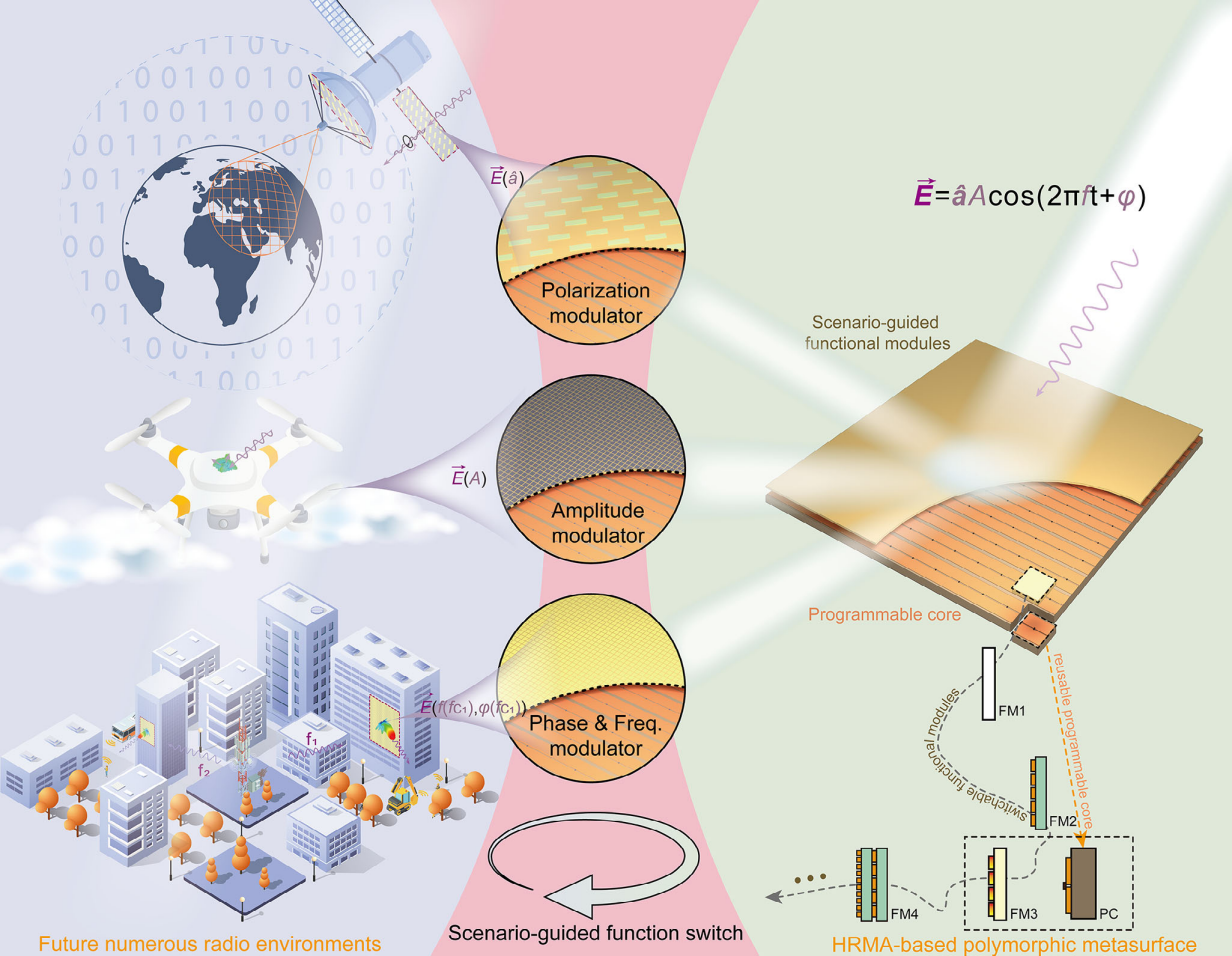
The proposed hierarchical reconfigurable metasurface architecture. The HRMA provides a new low‐cost solution for polymorphic functional metasurfaces. The PC is reusable across different scenarios, and the low‐cost FMs are designed according to the function and parameter requirements. The presented RMS can provide a platform for comprehensive EM scattering control, including flexible modulation of polarization, amplitude, phase, and frequency of EM waves. It provides a low‐cost and highly flexible solution for building intelligent wireless environments in the future.

A conceptual illustration of the proposed HRMA is depicted in Figure 1. A PC constructed by diodes, substrates, and bias structures is innovatively employed as a novel switchable load. FMs with low cost will be deliberately devised for specific scenario applications and parameters, and loaded onto the PC to build the target RMS. The PC enables spatial‐temporal modular encoding reconfiguration while serving as a critical component with hardware reusability. Specific FMs will endow metasurfaces with specific functionalities while physically protecting the PC, bringing super flexibility and stability to metasurface design. Considering the requirements of different scenarios, the regulation of EM wave fundamental parameters, including phase, frequency, amplitude, and polarization, can be obtained with the same PC and different FMs.

In a traditional paradigm, different RMS should be considered for different work scenarios, which brings huge production costs and cycles, while also introducing complex designs and significant instability. By sharing the PC and designing the FM with new functional and parameter requirements, the HRMA establishes a novel design paradigm, overcoming the conventional reconfigurable metasurface (CRMS) design constraints while enabling significant cross‐scenario ultra‐multi‐functional applications and transcending conventional design limits.

### 2.2 Programmable Core With Impedance Switching Flexibility

**FIGURE 2:**
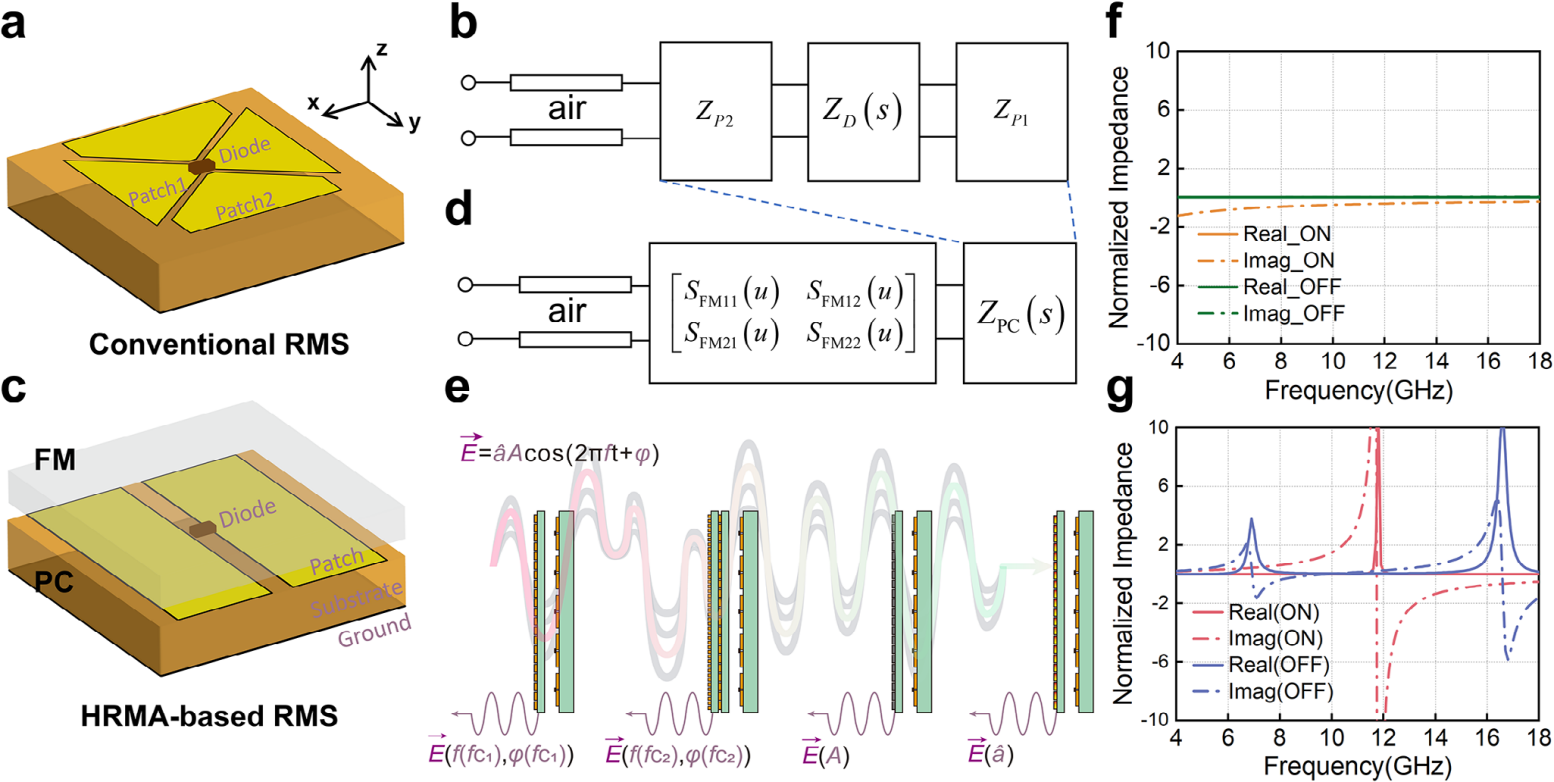
The difference between the traditional design method and the proposed scheme. (a) A traditional bow‐tie element for wideband phase modulation. (b) The equivalent circuit of the traditional RMS element during the design process. (c) The designed PC with FM mounted on. (d) The equivalent circuit of the proposed HRMA, which is constructed by a hierarchical cascade of FM and PC. (e) The schematic diagram of phase, frequency, amplitude, and polarization manipulation achieved through multiple FM switches. (f) The normalized impedance real and imaginary parts of commercial diode MADP‐000907 with 377 ohms as reference impedance. (g) The normalized impedance of the PC.

As the tunable components, diodes and phase change materials have been a key focus of attention in reconfigurable metasurface design for decades. Their reconfigurability endows the metasurface with vitality but also brings extremely high costs. Taking the PIN diode used in RF circuits for switching between ON and OFF states as an example, many studies have been conducted to manipulate the EM waves. As shown in Figure 2a, a typical bow‐tie element is constructed based on the traditional method. The diode is mounted between the bow‐tie shape with complex slots and parasitic structures for regulating the current path, generating resonance with specific functions. Typically, equivalent circuits with different impedances are cascaded before and after the PIN diode to achieve specific matching of the airport, as shown in Figure 2b. Under this paradigm, one scenario corresponds to one application requirement and also corresponds to the design and processing of a reconfigurable metasurface. Because the diodes have an inherent flaw related to the need for soldering, it will lead to high design costs and time.

Different from the traditional method, HRMA opens up a path for RMS from a new dimension. First, a novel reconfigurable load is designed and explored. As shown in Figure 2c, the diode, the metal shape for diode soldering and biasing, the substrate, and the ground plane are designed as the new load, namely programmable core. Besides, instead of complicating the structure around the diode, we achieve the matching design of the target function by adding a matching layer, called a functional module, at the front end of the reconfigurable load. Structurally speaking, the original reconfigurable layer has been simplified and retained, while a new stacked structure has been introduced for new dimension expansion. Therefore, the corresponding equivalent circuit model of the HRMA will be transformed into the structure shown in Figure 2d.

Consider only a single TE or TM incidence, the S‐parameters of a transmissive structure with a certain function assigned can be expressed as follows,

(1) $$S=\left[\begin{array}{cc} S_{11}\left(u\right) & S_{12}\left(u\right)\\ S_{21}\left(u\right) & S_{22}\left(u\right) \end{array}\right]$$

in which ‘*u*’ indicates the function implied in the structure and network. And for a reconfigurable reflective structure, its reflection coefficient Γ_PC_(*s*) in switchable states is closely related to its impedance.

(2) $$\Gamma_{L}\left(s\right)=\frac{Z_{L}\left(s\right)-Z_{0}}{Z_{L}\left(s\right)+Z_{0}}$$

When cascading the two components, we can find that

(3) $$\Gamma \left(s,u\right)=S_{11}\left(u\right)-\frac{S_{12}\left(u\right)S_{21}\left(u\right)\Gamma_{L}\left(s\right)}{1-S_{22}\left(u\right)\Gamma_{L}\left(s\right)}$$

Here, as depicted, the designed reconfigurable load is characterized with ZPC and connected to a functional matching network, which is designed to exhibit the network matrix of SFM, in which the former ensures reconfigurable state switching, while the latter flexibly provides polymorphic changes in functionality and parameters based on scenario requirements. Finally, the scattering coefficients of the metasurface will be observed from the airport.

(4) $$\Gamma_{\mathrm{RMS}}\left(s,u\right)=S_{\mathrm{FM}11}\left(u\right)-\frac{S_{\mathrm{FM}12}\left(u\right)S_{\mathrm{FM}21}\left(u\right)\Gamma_{\mathrm{PC}}\left(s\right)}{1-S_{\mathrm{FM}22}\left(u\right)\Gamma_{\mathrm{PC}}\left(s\right)}$$

Based on the architecture, the impedance switching and functional design are separated. And all the basic parameters of the scattering fields can be manipulated with one PC and switchable FMs, as shown in Figure 2e.

(5) $$E^{s}\left(s,u\right)=\Gamma_{\mathrm{RMS}}\left(s,u\right)E^{i}$$

In terms of impedance matching, for the design of RF circuits, diodes often achieve ON and OFF changes in a network with a reference impedance of 50 ohms. Here, as we are examining the scattering control of space waves, *Z*_0_ = 377 ohms is selected as the reference impedance. Figure 2f shows the normalized impedance results of a commercial PIN diode of MADP‐000907 type commonly used in traditional design. The real part is very small and close to 0 throughout the entire frequency band in both states, indicating this diode's low‐loss property. The imaginary part shows a negative value in the OFF state, increasing with frequency from 4 to 18 GHz. Therefore, intuitively speaking, considering diodes as reconfigurable devices is a continuation of traditional RF circuit design to spatial electromagnetic wave device design. Obviously, for the regulation of spatial scattering waves, diodes no longer exhibit significant switching state changes, but rather a certain degree of impedance differences. Therefore, for the acquisition of multiple typical scattering characteristics in common designs, this reconfigurable load may no longer be optimal.

In the new paradigm, the PC is employed as the reconfigurable load. Here, a simple microstrip connection line type reconfigurable element is selected as the PC, with its parameters kept unchanged and its structure reusable in the work. Here, for functional demonstrations and subsequent design needs, the PC is first designed using traditional methods with a 1‐bit phase modulator as the target during construction. The specific design process and structure parameters are given in Note S1. Finally, its impedance is obtained as depicted in Figure 2g. As can be seen, Figure 2g shows the impedance of the PC in HRMA design with characteristic impedance 377 ohms. For simplicity, the PC designed here has impedance reconstruction properties in ***x***‐polarized incidence (TM modes), but no reconstruction design has been made for ***y***‐polarized incidence (TE modes). Under TE incidences, PC shows a structure similar to a connected ground, with the real part of impedance being 0 and the imaginary part being extremely small. For TM incidences, the reconfigurable load is neither a simple switching state presented by the switches in the circuit nor a weak capacitive inductive change but rather exhibits obvious resonance phenomena. For this PC load, a resonance point appears at 11.7 GHz with the diodes in the OFF state, and two resonance points appear at 7 and 16.4 GHz in the ON state. It will be found that a solid foundation for subsequent functional module design is established to achieve corresponding applications with these initial resonance points.

Through this architecture, three huge advantages will be brought. First, new reconfigurable loads no longer require welding and can be flexibly reused. Second, the addition of the top layer forms a new dimension of regulation, promoting flexible function design. Therefore, there is a possibility of separation between functional implementation and load switching. Third, the top layer will provide protection for the diode and enhance the robustness of the metasurface.

### 2.3 Design of Phase and Frequency Modulators With Extreme Spectrum Width

The past decade has witnessed a proliferation of 1‐bit phase‐modulating metasurfaces and their derived applications, while traditionally, the operational bandwidth remains fundamentally constrained by the inherent narrowband characteristics of semiconductor diodes' dispersion impedance and the structural resonance limitations. HRMA provides a new approach and perspective.

**FIGURE 3:**
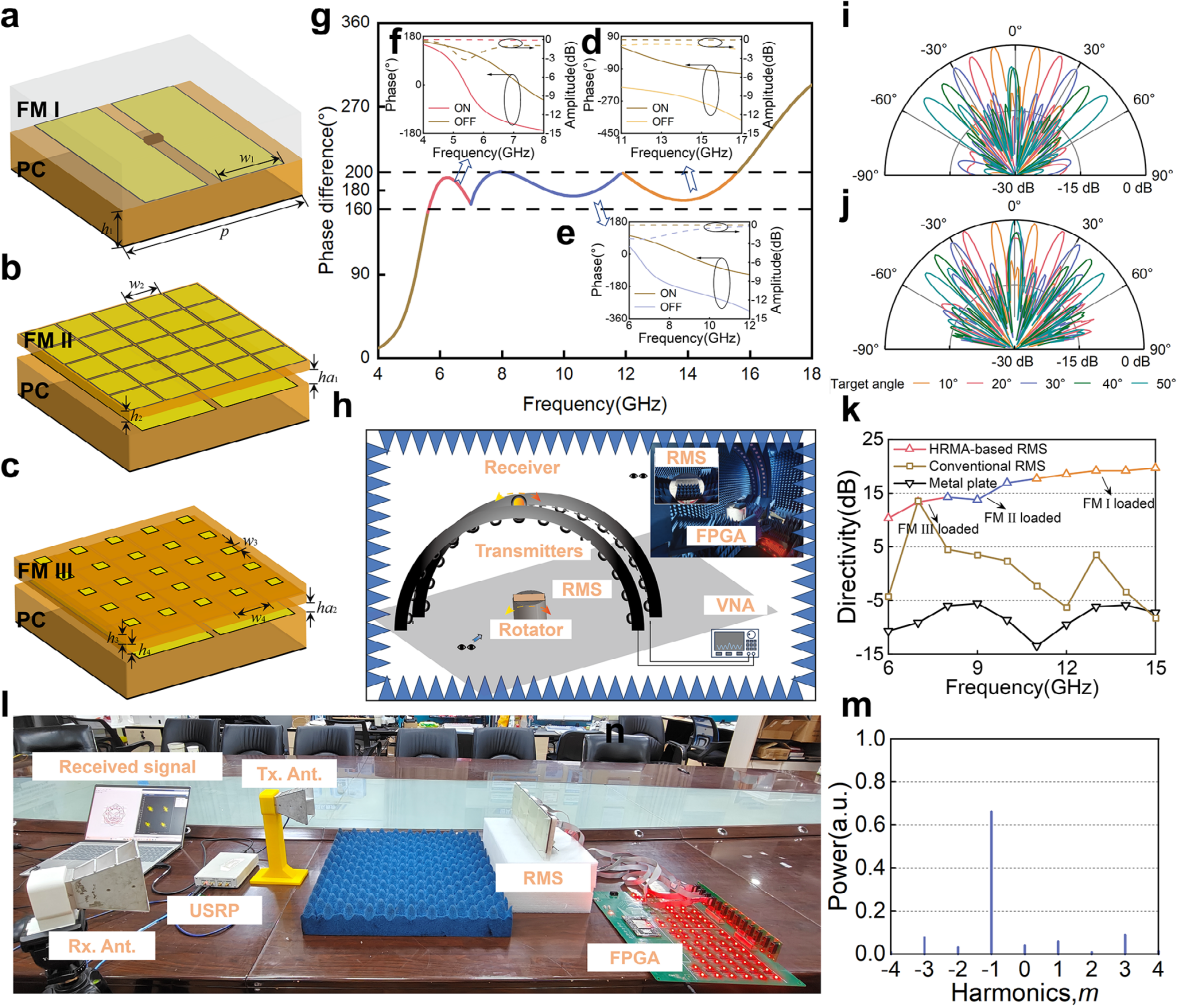
The construction and results of the phase and frequency modulator based on HRMA. (a–c) The element structure with FM I‐III loaded on PC. (d–f) The simulated reflection phase and amplitude curves corresponding to the elements in a–c. (g) Comprehensive 1‐bit phase modulation curves of three types of RMS. (h) The test environments of the metasurface in a chamber. (i,j) The simulated and measured RCS patterns of the RMS with FM II loaded. (k) The comparative simulation results of directional coefficients for HRMA‐based RMS, conventional RMS, and metal plate at a target beam direction of 50°. (l) The construction of a communication system with FM I loaded RMS. (m) The measured pattern harmonic manipulation results of the RMS working as a frequency modulator with FM II loaded, to manipulate the −1^st^ harmonic to the target angle of −23°.

As detailed in Note S1, the PC is optimized first when an air layer is selected as FM I, since the air layer is a natural FM of PC. And finally, the formation of a Ku‐band phase modulator is achieved at 11.8–15.5 GHz, as shown in Figure 3a, and its reflection phase results in Figure 3d. In fact, from our perspective, traditional methods consider the air layer as the functional module and explore the function and parameter limits of the metasurface through complicated designs of the metal structures around diodes.

The FM can be designed as needed and flexibly switched, while the PC can be reused. Through this method, the parameter limits of metasurfaces will be extended. For demonstration, another two FMs are designed. A fast analysis method is detailed in Note S2. Constructed by a dielectric layer with a periodic subwavelength square patch layer mounted on, FM II macroscopically constitutes the equivalent medium with an effective permittivity of 4.17 + 0.53i and permeability of 0.82 + 0.42i at 10 GHz as described in Note S3. It should be noted that the diodes on the PC have a thickness of 0.3 mm, and thus, a certain air thickness needs to be considered when designing the FM. Here, FM II includes the 1 mm air layer above the PC. Here, through the design and loading of FM II, a new phase modulator is formed as depicted in Figure 3b and the results shown in Figure 3e. At this time, the RMS also maintains 1‐bit phase modulation capability, but its operating frequency has changed from Ku‐band to X‐band, covering 6.8–11.9 GHz. Similarly, when further switched to FM III with an effective permittivity of 12.92 + 0.05i and permeability of 0.95 + 0.004i, another new phase modulator operating in C‐band covering 5.6–7 GHz is generated, of which the structure and reflection phase are given in Figure 3c,f.

As described above, in the case of reusing the same PC, we have achieved the operational spectrum switching of C‐, X‐, and Ku‐bands. Here, three FMs are deliberately designed to achieve the continuity of the three working frequency bands. In other words, we have designed a 1‐bit phase modulator that switches the functional module and covers 5.6–15.6 GHz as shown in Figure 3g, which significantly surpasses the bandwidth limitations of the proposed broadband metasurfaces. It should be noted that although three typical FMs and the working frequency band results of metasurfaces are provided as examples, in fact, we can match the impedance of other frequency bands of reconfigurable metasurfaces through the design of FMs, constructing 1‐bit phase modulation metasurfaces at the target band flexibly.

Differences manifest not only in the element design method and the reflection response. When the elements are assembled into an array, the significant features and advantages of the proposed HRMA architecture should also be emphasized. Since the FM can be switched and the PC can be agile, the metasurfaces array exhibits flexible modulation in both the spatial and temporal domains. Here, a 12 × 40 array is constructed for design validation of the metasurface on array level, simulations are conducted in Ansys Desktop 2023 R2 and measurements in a microwave anechoic chamber as shown in Figure 3h. Calculated by the diffraction grating equation detailed in Note S4, Figure 3i,j shows the simulated and measured results of 1‐bit quantization of the phase distribution of 10°–50° beams under 0° incidences at 10 GHz. Efficient deflection manipulation can be achieved arbitrarily under the switching of the encoding pattern. Deflection covering ultra‐wideband, which traditional RMS is almost impossible to reach, can be observed. As shown in Figure 3k, compared to metal plates of equal size, traditional metasurfaces can achieve a directional coefficient improvement of about 20 dB at a target angle of 50° near their operating frequency of 6 GHz. However, when the operating frequency exceeds their coverage bandwidth, the improvement gradually weakens and approaches the effect of metal plates. While based on HRMA, our RMS can break through this constraint with the same PC. In order to achieve meticulous deflection control covering ultrawideband frequency bands with a large angle of 50°, it is necessary to redistribute the encoding pattern according to the operating frequency and switching FM. The calculated pattern and the information of the conventional RMS are depicted in Figures S7 and S8. As shown in Figure 3k, the beam‐steering capability of the proposed RMS at a target angle of 50° exhibits stable and excellent performance, with directivity gradually increasing as the operating frequency rises. This demonstrates the significant advantages of HRMA‐based metasurfaces over traditional solutions in practical applications. We only need to design one PC and multiple FMs at low cost to achieve 1‐bit modulation coverage for ultra‐wideband applications, including RIS communication, stealth applications, and so on. Besides, it should be noted that, based on this scheme, it also has great advantages in the design of low‐interference RIS [30], because multiple frequency bands can be implemented using different FMs separately. Further, for the application demonstration, an auxiliary communication experiment based on this metasurface is constructed in Figure 3l at 6 GHz. The communication system constructed using Universal Software Radio Peripheral (USRP) and antennas effectively achieves stable image transmission through channel modulation based on the metasurface. In fact, with the ability to control channels covering the C‐, X‐, and Ku‐bands, we can achieve channel manipulation covering ultra‐wide frequency bands using only one PC and three different FMs through the layout and design of this metasurface.

At the same time, the RMS also possesses the capability of frequency modulation through spatiotemporal modulation technology. Figure 3m presents the results of the ‐1st harmonic manipulation with the 10 GHz monochromatic wave incidences. The modulation rate of the FPGA is set to 500 kHz, and the adopted space‐time matrix is given in Figure S9, and the design method is detailed in Note S5. By using space‐time coding modulation, the conversion of fundamental energy was successfully achieved, and the ‐1st harmonic wave is efficiently manipulated to −23°. And according to space‐time coding theory, efficient frequency modulation can be achieved at any 1‐bit operating frequency. That is to say, bandwidth extension is also effective for frequency modulation.

### 2.4 Amplitude and Polarization Modulator Design for Polymorphic Function Switch

When the demand for metasurfaces comes to stealth applications, due to the fixed nature of the limited functionality of traditional RMS, different requirements for operating frequency bands and functions, the previous design and processing will inevitably be overturned. The proposed HRMA has potential functions and applications in more dimensions with the reuse of the PC. Here, the amplitude manipulation of EM waves is demonstrated further by simply switching the FM.

**FIGURE 4:**
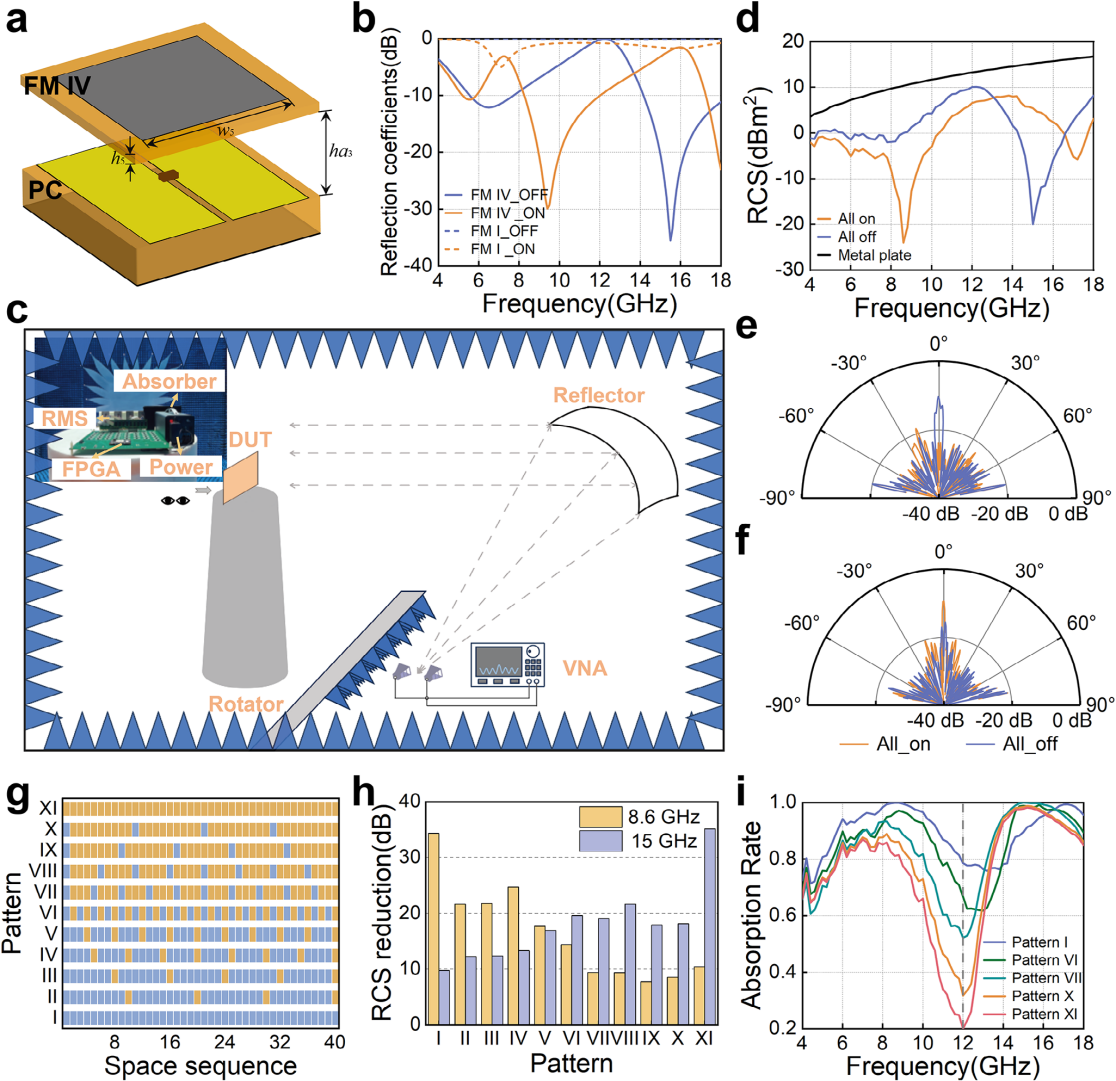
The performance of the reconfigurable absorber designed based on the proposed scheme with the PC and FM IV. (a) The construction of the element with FM IV mounted. (b) The simulation reflection coefficients of the element under normal incidences of ***x***‐polarization. (c) The testing mechanism and environment for single station RCS measurement in a compact chamber. (d) The measured RCS of the prototype and metal plates of equal size under normal incidences. The simulated and measured scattering patterns at 8.6 and 15 GHz when PCs are fully (e) off, and (f) on. (g) The measured pattern for tunable absorption. (h) Tunable RCS reduction value compared to the metal board at 8.6 and 15 GHz in the given patterns. (i) The absorption rate tunability of the specific patterns at 12 GHz.

FM IV is designed to equip the metasurface with the ability of amplitude regulation and transform it into a dual‐band switchable absorber, as shown in Figure 4a. The design and construction are detailed in Note S6. Different from phase modulators with FMs I‐III, new resonant poles are formed within the frequency band after adding this layer, and the reflection amplitude decreases sharply in Figure 4b. Throughout the frequency range, electromagnetic energy is absorbed by the metasurface in this case. Obviously, FM IV has caused significant changes in the overall working attributes of the metasurface, shifting from phase control to amplitude control. It is also worth noting that we reuse the PC again. Besides, when the PC makes a ON/OFF state switch, different responses can also be triggered at target frequencies. That is to say, not only has the basic functionality changed, but the reconfigurable properties are still retained, and regulatory flexibility is introduced in the design.

Here, as an example, two design frequencies commonly used for stealth applications at X‐ and Ku‐bands are selected. From the element level, the reflection coefficients illustrate the change in function distinctly. Specifically, for TM incidences, when the PC switches to the ON state, the element forms an absorption state with the reflection coefficients below ‐10 dB at 8.3–11.9 GHz, and when the state switches to the OFF state, the main absorption range is moved to the 14.2–18 GHz frequency band. At 9.5 GHz in the ON state and 15.5 GHz in the OFF state, the reflection coefficients reach a minimum below −30 dB. Here, the reflection coefficients when FM I is loaded are provided for comparison. By switching FM, the functionality of metasurfaces has achieved a leap in changes. Besides, it is also worth mentioning that the designed reconfigurable absorber forms an amplitude modulator with an amplitude difference greater than 10 dB in the 8.5–12 and 14.5–16.5 GHz frequency bands, making it also a promising broadband amplitude modulator working at dual band for a communication system based on a spatial electromagnetic wave amplitude modulator.

The metasurface array with FM IV mounted is measured in an anechoic chamber with compact range, as depicted in Figure 4c. The monostatic RCSs under different states are measured. First, the monostatic RCS levels at broadside incidences in the frequency domain are observed and analyzed in Figure 4d when the PCs are turned on and off as a whole. Despite some frequency offset caused by assembly and processing factors, the simulation and actual measurement results maintain a high degree of consistency. The measured RCS results of the metal board of equal size are provided for comparison. And the results show that for TM polarization, when the PCs are turned off, the measured RCS level exhibits a minimum pole formed around 15 GHz, and when in the ON state, the pole switches to around 8.6 GHz. That is to say, when a hostile target is detected operating in the X‐band, the PCs can switch to a fully ON state to maximize the stealth performance of the X‐band. At the same time, when the target is a Ku‐band radar, the optimal stealth performance of the target is achieved in a fully OFF state. At 8.6 and 15 GHz, under ***x***‐polarization excitation, the monostatic scattering pattern of the metasurface during ON and OFF state switching is shown in Figure 4e,f. Simulation and experimental results show that within the ±2.5° angle domain, the energy is significantly absorbed by the metasurface. In the measured results, the jitter of the RCS level at large angles is caused by testing errors generated by the control circuit board and power devices.

When the FM is fixed, flexible control is still accessible in the PCs. In Note S7, manipulation for RCS level and absorption rates at key frequency points is explored. There are 11 patterns measured as listed in Figure 4g, and the measured RCS reduction values compared to the metal board at 8.6 and 15 GHz are depicted in Figure 4h. As stated in Note S7, with the change of the proportion of ON and OFF state elements in the PCs, significant changes in RCS reduction values are observed at the two key frequency points. As the proportion of elements in the OFF state increased, the reduction at 8.6 GHz increased overall, while conversely, the reduction at 15 GHz increased. This indicates that RCS reduction values at specific frequencies can be specifically designed through the regulation of PCs. Figure 4i shows the overall absorption ratio across 4–18 GHz. Through various coding designs, the metasurface has achieved above 0.7 at all frequencies. Besides, when adopting the above different patterns, significant changes in absorption rate were also observed at 12 GHz. The absorption rates can be tuned from 0.2 to 0.8 as depicted. The HRMA‐based reconfigurable absorber exhibits excellent broadband absorption characteristics and state switching capability. Furthermore, it should be noted that, in order to achieve scene adaptive absorber design, FM IV can also be replaced at low cost, achieving flexible implementation of target parameters.

As introduced above, by reusing the PCs and solely reconfiguring the FM, the phase modulator is dynamically morphed into a dedicated amplitude modulator, enabling RCS modulation in the target band. Further, polarization, another basic parameter of electromagnetic waves, is also verified to be regulated through this architecture. Here, the design of a Ku‐band linear polarization converter is further taken as an example for demonstration, since the frequency band around 14 GHz is commonly used for satellite communication.

**FIGURE 5:**
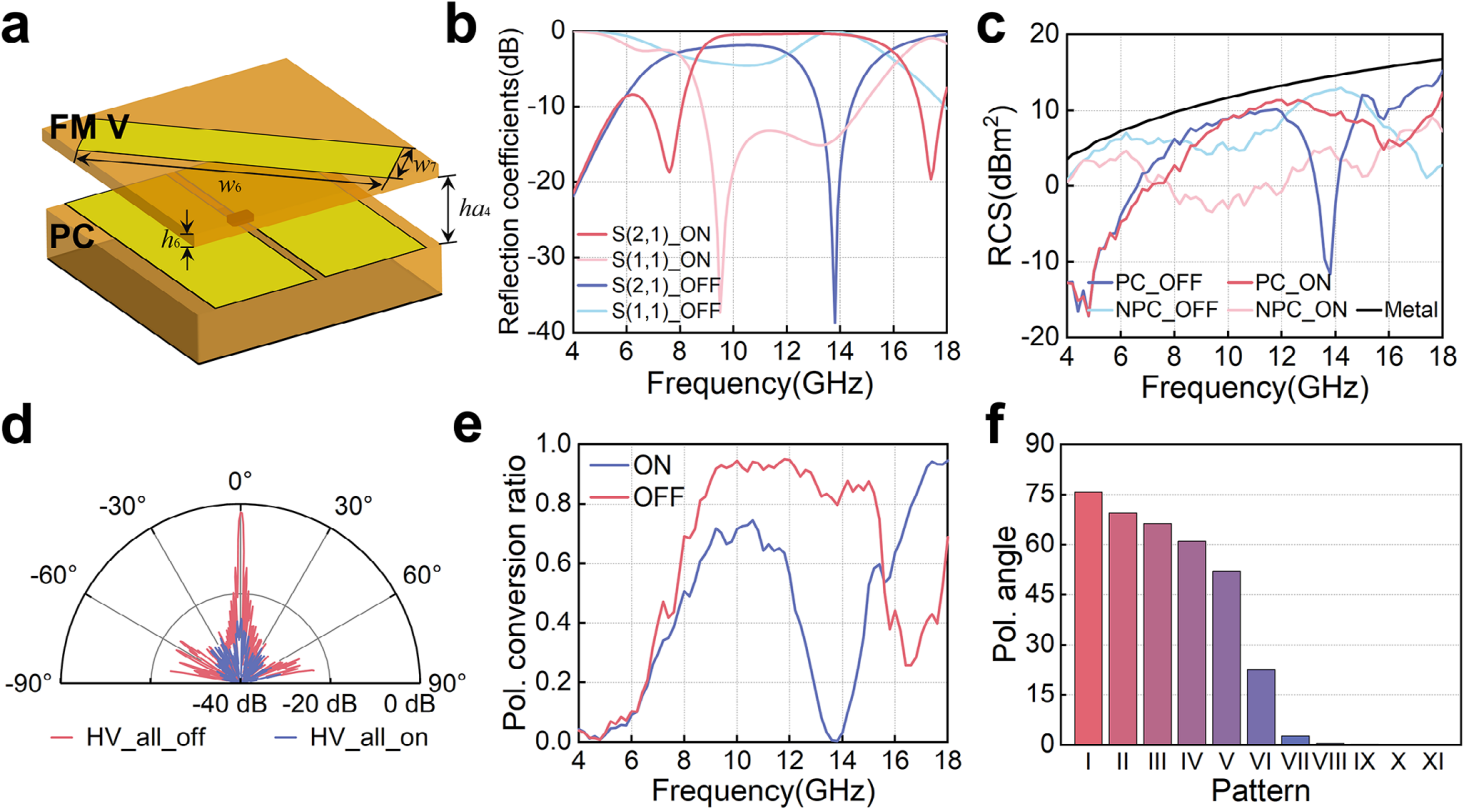
The performance of the reconfigurable polarization converter designed based on the proposed scheme with the PC and FM V. (a) The construction of the element with the FM V. (b) The simulation reflection coefficients of the element in periodic boundaries. (c) The measured monostatic RCS results of the prototype at broadside incidence, in which ‘PC’ means the polarization conversion components and ‘NPC’ the non‐polarization conversion components. (d) The measured RCS angular pattern results at 13.8 GHz. (e) The measured polarization component ratio when the PCs are all turned on and off. (f) The polarization angle when patterns with different ratios are adopted.

At this time, FM V adopts a rectangular patch oriented at 45° as depicted in Figure 5a. The polarization conversion component of FM V cannot be ignored, forming a bi‐anisotropic medium, which is detailed in Note S8. The polarization conversion component and reflection coefficients of both TE and TM modes are first analyzed. As given in Figure 5b, a broadband orthogonal linear polarization converter is formed when FM V is replaced and PCs are turned on. The polarization conversion coefficients maintain more than −1 dB from 8.8–14.8 GHz, which means a polarization conversion efficiency greater than 90%. Due to the reconfigurable properties of PCs, metasurfaces can also achieve co‐polarized reflection at 13.8 GHz in the OFF state. With FM V mounted, the metasurface can not only flexibly achieve broadband polarization conversion, but also switch off the polarization conversion to reflect the co‐polarized component.

For the verification, FM V is processed and laid out above the PCs, constituting a polarization modulator. The reconfigurable polarization converter is measured in the measurement environment as shown in Figure 4c. Here, to evaluate the polarization conversion capability, the metasurface is placed along horizontal polarization, and the scattering levels of horizontal and vertical polarization were measured under horizontal polarization incidence. Figure 5c depicts the measured results with all the PCs turned on and off. The measurement results show high consistency with the element results in periodic boundaries. As is depicted, a significant polarization conversion is observed from 8.9–14.7 GHz in the ON state. Besides, when the PCs are all switched off, a total reflection band of co‐polarization component around 13.8 GHz is generated. This means that the energy within the frequency band is almost not converted, and through the encoding switching of the PCs, polarization conversion and non‐conversion states can be achieved. Figure 5d shows the angular pattern of the monostatic RCS results at 13.8 GHz, which illustrates that when approaching broadside incidences, the metasurface experiences two completely different states of polarization conversion and non‐conversion. The polarization component ratio is then calculated and given in Figure 5e. In fact, the PCs still maintain higher encoding flexibility, promoting more flexible polarization conversion. With the distribution of ON and OFF states in different proportions of the PCs, the polarization angle can also be designed arbitrarily within the range of 0–90°. Figure 5f shows the measured polarization angle of 11 patterns at 13.8 GHz. The polarization rotation angle changes from 0°–75°. Although some polarization angles are not shown, it can be inferred that this polarization conversion metasurface can cover all polarization angles when the scale of the metasurface is large enough and a mirrored FM V is adopted. Detailed explanations are provided in Note S9. Besides, it should be noted that though only the converter of linear polarization is demonstrated here, many other polarization devices, including linear to circular polarization converter, or vice versa, can be designed and achieved with different types of FMs.

### 2.5 Design and Performance Comparison of HRMA‐Based RMS and CRMS

A comparison of the functional RMSs and the designed HRMA‐based RMS is further implemented. As detailed in Table 1, the HRMA endows the designed metasurface with the potential for function expansion and parameter adjustment, which makes it more competent for versatile and multi‐scenario metasurface designs in the future.

**TABLE 1:** Comparison of the state‐of‐the‐art RMSs and the proposed HRMA‐based RMS.

Reference	Demonstration of wave manipulation capability				
	Phase	Amplitude	Frequency	Polarization	Parameter adjustment
[13]	√	√	×	×	×
[15]	√	√	√	×	×
[17]	√	×	√	√	×
[38]	√	×	×	×	×
[39]	√	√	×	×	×
This work	√	√	√	√	√

**FIGURE 6:**
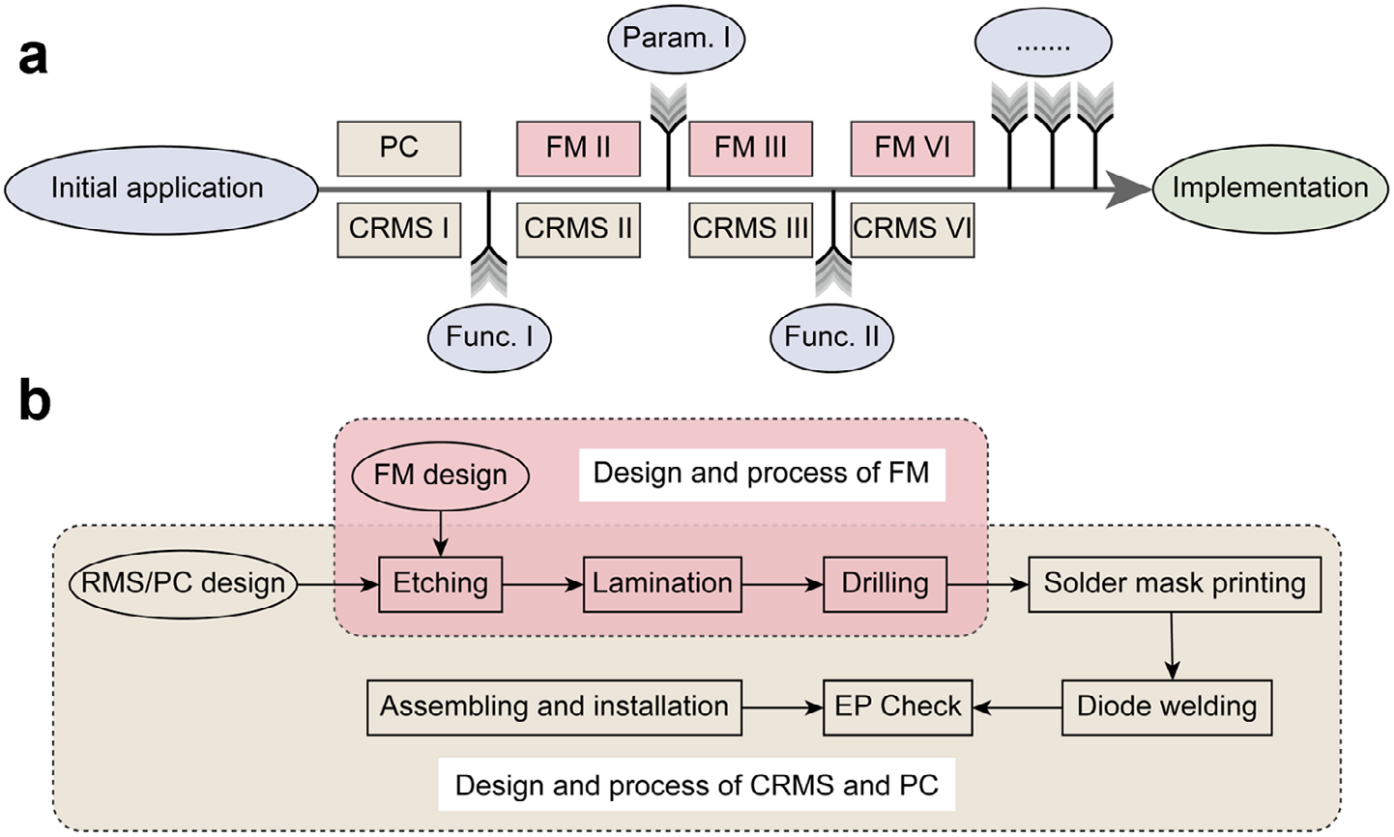
The design process of the HRMA‐based RMS compared to the CRMS. (a) The design scenario with functions and parameters adjustment. (b) The design and process of the PC, FM and CRMS.

HRMA has brought great help to the multifunctional realization of the metasurface. It is slightly different from the design of the CRMS, but basically maintains similar performance. The design process of the HRMA‐based RMS and CRMS under multiple function switching is depicted in Figure 6. As is introduced above and in Figure 6a, PC and CRMS can be designed when the initial application is given. However, when the required functions or parameters vary, the need for thorough redesign and processing can not be avoided for the CRMS. For the proposed HRMA, only the FM is required to be redesigned and processed according to the load impedance of the PC and the new functions and parameters. Throughout the process, for the demand changes, HRMA only requires one PC processing, involving complex processes and high costs similar to the CRMS design. In the subsequent demand response, only the design and processing of FM need to be considered, which will significantly reduce the processing time and costs. Specifically, Figure 6b illustrates the benefits HRMA brings. For the CRMS process, etching, lamination, drilling, solder mask printing, diode welding, electrical performance (EP) check, assembling, and installation need to be implemented step by step, while FM processing only requires the first three steps. It is worth noting that this method of separating FM and PC also tends to make the structure simpler than traditional multifunctional metasurfaces, as CRMS needs to meet more requirements in one structure. In the work, the PC and FMs are assembled through fixing holes and screws, and the assembly process can lead to errors and affect the performance of the RMS. The possible errors and their impact on the proposed RMS performance are analyzed and evaluated in Note S10.

The costs of the proposed HRMA‐based RMSs are also provided in Table 2 for the illustration of the help of HRMA in cost reduction. As can be seen, the material and processing costs of PC account for a significant portion, with the main cost coming from 480 MADP‐000907‐14020w diodes covering the microwave frequency band, with a unit price of ¥21.18 [45]. The material and processing costs of FM are very low because it does not require any complex processes. The cost of substrate material is calculated based on the common 20 × 24 inches standard, however, this is much larger than the size of the verified metasurface array, indicating that the cost of RF substrate materials will be further lower involving large‐scale processing, which will particularly reduce the material cost of FMs. In terms of processing time, PC processing has also become the main contradiction. The assembly line flow of PCB processing will affect its progress, while FM only requires simple etching and other operations, which can be completed within a few hours.

**TABLE 2:** Processing cost and time of the proposed PC and FMs.

Item		PC	FM I	FM II	FM III	FM VI	FM V
Cost(¥)	Material	10870	∖	253	438	270	270
	Processing	3631	∖	486	972	486	986
	Total	14501	∖	739	1410	756	1256
Processing time		8 days	∖	<1 day	<1 day	<1 day	<2 days

For the five functions and parameters mentioned, the processing cost of PC in HRMA‐based RMS design accounts for 100%, 95%, 91%, 95%, and 92%, while the processing time accounts for 100%, 89%, 89%, 89%, and 80%. These applications can also be implemented through CRMS, however, RMS with any functionality achievement is almost impossible to achieve. Designing five targeted RMSs will lead to a surge in costs and time costs. Estimating CRMS at the same cost and time as PC, HRMA‐based RMS only accounts for 25% of the CRMS cost and 32.5% of its time consumption in terms of the proposed features, which will be more significant in terms of more functional requirements and switching.

Finally, it should be noted that HRMA itself does not cause performance degradation. The designed RMS, consisting of PC and FM I‐III demonstrate a wide phase bandwidth and maintain low loss characteristics compared to the existing RMS for RIS applications [46]. The reconfigurable absorber and polarization converter also demonstrates good bandwidth, absorption rate, or polarization conversion rate compared to existing designs [47, 48]. Although the designed absorber does not show a particularly prominent bandwidth, the bandwidth expansion is expected to be achieved through FM design [49]. In fact, compared with the traditional scheme, the defect of HRMA is the increase of profile height. Because the impedance matching structure is not designed on the horizontal plane of the PIN diodes, but on the FM layer on the top of the PC. However, in many scenarios, such a profile is acceptable.

In principle, PC can be any form of switchable impedance load, while FM is any form of impedance converter that can transform the impedance at the PC end to the target function and parameters as needed. Currently, FM is often composed of low‐loss RF substrates, which hardly introduce additional losses. HRMA‐based RMS is actually a compromise between single requirement, simple design, good performance, and any requirement, complex design, and average performance of CRMS. In scenarios where there is no need for quick functional changes, HRMS‐based RMS will inject new vitality into the multifunctional design, low‐cost processing, and recycling of RMS.

## 3 Conclusion

In summary, a novel and flexible hierarchical reconfigurable metasurface design architecture for the expansion of scattering manipulation reconfigurability is proposed. The functional regulation of scattering has become a new dimension being explored. The metasurface is modularized and layered into functional modules and a programmable core, which can be designed separately. The method does not consider PIN diodes as reconfigurable terminals, but instead treats the programmable core with diodes as a novel and reusable reconfigurable load. With the PC reused, mutable functional modules can be designed and switched, expanding working parameters and application functions according to the needs of the scenario. Independent of the functional module, the PC maintains the degree of control freedom in both spatial and temporal dimensions, retaining the inherent diversity and advantages of RMS in coding design in existing work.

Three types of functional reconfigurable metasurfaces are designed with a shared PC, manipulating all the basic parameters of EM waves, including phase, frequency, amplitude, and polarization. Based on this design method, the phase modulators form a 1‐bit phase resolution bandwidth working from 5.6–15.6 GHz which far exceeds existing metasurfaces and can be continuously expanded. With the scenarios changing, the functional modules can also be changed whenever necessary. Flexible amplitude and polarization modulators working at X‐ and Ku‐bands are designed with simple FMs switched, achieving tunable maximum absorption peak with 99% efficiency and high absorption in X/Ku‐bands (8.4–12 GHz/14.5–16 GHz) with 90% efficiency, and switchable linear polarization conversion at Ka‐band (14 GHz).

From the demonstrations verified above, we can clearly see the significant advantages of this design method in breaking through parameter limits and expanding multifunctionality. Here, the architecture is used in the design of the most commonly used electronically controlled PMS, while it should be noted that this proposed HRMA architecture demonstrates universal applicability across electronic, optical, acoustic, magnetic, and mechanical controlled RMS paradigms. New chips can be designed for scattering control based on the proposed PC and FMs. Through this method, improvements can be made in dimensions such as cost, flexibility, functionality, and parameter limitations in the design and application of spatiotemporal modulation metasurfaces, intelligent reconfigurable metasurfaces, etc.

## 4 Experimental Methods

### 4.1 Prototype Design

By verifying the characteristics of the element in an infinite periodic boundary, we have obtained preliminary results. When the array size is large enough, edge effects and local resonance phenomena can be ignored. The detailed design method of the element is described in the Supporting Information. All diodes are arranged in the same direction along the *x*‐axis. Uniform bias are applied to the 12 elements in each column. An inductor (LQW15AN7N0G00) is selected to isolate DC and RF at the end of each column. Within the design frequency band, the *S*_21_ isolation is greater than 10 dB. When the PIN diodes are turned off with a biasing voltage of −5 V, the PC element is in OFF state with is impedance shown in Figure 2g. On the contrary, the element is ON state as the PIN diodes are turned on with a forward biasing voltage of +5 V. To bias the 48 column of the PC separately, an FPGA control board is designed using the commercial PCB Design software, Cadence Allegro. The FPGA AV7K325 is used as the core of the control circuitry. A precision quad SPDT analog switch, DG333ALDW, is selected for the bias of the array. The SPDT Each output channel of the chip is connected in series with a 330 Ω current‐limiting resistor to set the current of the PIN diode on the unit to 10 mA. Meanwhile, the control board is equipped with LED beads for monitoring the output status of each channel.

### 4.2 Prototype Simulations

To study the scattering characteristics of the metasurface, simulations at the element and array levels are conducted in Ansys Desktop HFSS 2023 R2 for the proposed model. When evaluating the element, we assign periodic boundaries and a Floquet port to observe the performance of the element in an infinite period. The PC is modeled as a load, of which the impedance is calculated from the *S*‐parameter file extracted from the simulations in Note S2. For the array level, FE‐BI boundaries are assigned to the air box, and a plane wave excitation perpendicular to the array is set to observe the RCS characteristics of the array.

### 4.3 Prototype Fabrications

Two commercially available S7136H substrates (relative dielectric constant *ε*_r_ = 3.55; loss tangent tan*δ* = 0.002) with a thickness of *h*_1_ = 2.012 and 0.254 mm were used as the substrates of the PC, which are bonded by a Rogers RO4450F prepreg with εr = 3.52, tanδ = 0.004 and a thickness of 0.10 mm. The functional module III was constructed by S7136H substrates with *h*_3_ = 0.254 mm and *h*_4 = _0.508 mm, and functional module II, IV, V by F4BM substrates (relative dielectric constant *ε*_r_ = 2.2; loss tangent tan*δ* = 0.009) of *h*_2_ = 0.508 mm, *h*_5_ = 1.016 mm, and *h*_6_ = 1.016 mm. The modules were fabricated using a commercial printed circuit board manufacturing process. When processing functional module III, the metal on both sides of the copper‐clad laminate was first etched, and then resistive ink was sprayed on the top. The PIN diodes (MACOM MADP‐000907‐14020x) were added on top of each loop slot by reflow soldering. To achieve a specific air layer thickness, nylon pads with thicknesses of *ha*_1_ = 1 mm, *ha*_2_ = 0.3 mm, *ha*_3_ = 4 mm, and *ha*_4_ = 2 mm are used as support for the functional module as needed. The other parameters are *w*_1_ = 3.35 mm, *w*_2_ = 1.45 mm, *w*_3_ = 0.5 mm, and *w*_4_ = 1.5 mm.

### 4.4 Prototype Measurements

The fabricated metasurfaces were characterized inside microwave anechoic chambers, the measurements of bistatic RCS in a double probe arch frame anechoic chamber, and monostatic RCS in a compact field anechoic chamber, respectively (Figures 3m and 4c). The scattering bistatic RCS pattern measurement of the metasurface was carried out in a multi‐probe microwave anechoic chamber, with 31 receiving antennas arranged along an arc‐shaped array connected to a VNA (Ceyear 3674D). A transmitting antenna that can be moved at any angle along the arc can adjust the incident angle of electromagnetic waves. At the same time, the turntable on which the metasurface is placed can also be tilted at any angle. In order to measure the scattering pattern of the metasurface at 0° incidence, the turntable is tilted at intervals of 1° from 0° to 5° during testing. The transmitting antenna is also rotated at the same angle along the arc to obtain the scattering field. By employing background cancellation, RCS standard calibration, and near‐field extrapolation methods, a bistatic RCS with 0° incidence was ultimately obtained. During monostatic RCS measurements, a 3D printed bracket of 120 × 320 mm was designed for fixing and installing metasurfaces during the measurement. The FPGA control board was placed horizontally on the back side of the metasurface during testing for biasing of the coding layer. A portable power source was used to supply power to the control board and placed behind a QYH‐P20 absorber with a thickness of 20 mm and 25 dB absorption. Two linearly polarized horns were applied as the receiving (Rx) and transmitting (Tx) antennas connected to a vector network analyzer (VNA, Keysight N5244B) to detect the scattering signal level. A reflector is placed about 6 m away from the test prototype, and the distance between the antennas and the reflector is 6 m, ensuring that the measurement meets the far‐field condition.

## Conflicts of Interest

The authors declare no conflicts of interest.

## Supporting information




**Supporting File**: advs74738‐sup‐0001‐SuppMat.docx.

## Data Availability

The data that support the findings of this study are available from the corresponding author upon reasonable request.
