# Supplementary material for: Hierarchical Reconfigurable Metasurface Based on Scenario‐Guided Functional Modules and Programmable Core
Source: Adv Sci (Weinh). 2026 Mar 13;13(28):e24154. doi: 10.1002/advs.202524154 (PMC13185813; doi:10.1002/advs.202524154)
Supplement: Supplementary file 1 — Supporting File: advs74738‐sup‐0001‐SuppMat.docx. [file ADVS-13-e24154-s001.docx]

Supporting Information

**Hierarchical reconfigurable metasurface based on scenario-guided functional modules and programmable core**

Lihao Zhu, Jiaqi Han, Zhe Zheng, Qiang Feng, Dexiao Xia, Xiangjin Ma, Yajie Mu, Guoliang Luo, Rui Li, Haixia Liu, Hao Xue*, and Long Li*

Key Laboratory of High-Speed Circuit Design and EMC, Ministry of Education, School of Electronic Engineering, Xidian University, Xi'an 710071, China

*E-mail: [xuehao@xidian.edu.cn](mailto:xuehao@xidian.edu.cn) and [lilong@mail.xidian.edu.cn](mailto:lilong@mail.xidian.edu.cn)

This supplementary information includes:

Supplementary Note 1. The parameter determination and properties of the PC

Supplementary Note 2. Fast analysis and design of metasurface elements based on HRMA

Supplementary Note 3. Design and configuration of FMs Ⅰ-Ⅲ for an ultra-wideband phase modulator

Supplementary Note 4. Beam modulation based on the space wave diffraction grating equation and generalized Snell’s law

Supplementary Note 5. Frequency modulation based on space-time-coding metasurface

Supplementary Note 6. Design and configuration of FM Ⅳ for a broadband reconfigurable absorber

Supplementary Note 7. The synthesis of an adjustable absorber with the capability of tunable RCS modulation depth

Supplementary Note 8. Design and configuration of FM Ⅴ for a reconfigurable polarization converter

Supplementary Note 9. The synthesis and realization of a linear polarization converter with any polarization angle

Supplementary Note 10. Analysis of the impact of assembly errors on the performance of HRMA-based RMS

Supplementary Note 1. The parameter determination and properties of the PC

Unlike the traditional design process, the construction of PC should be considered, in which functional designs are conducted. For PCs containing PIN diodes, the loading and bias methods of the PIN diodes need to be considered. For simplicity, a basic connected microstrip scattering metasurface element is adopted in the design. The rectangular patch extends infinitely in the ***x***-direction and is periodically slotted in the y-direction. In the slot at the center of the element, a PIN diode is periodically loaded for the coding reconstruction design of the metasurface. Without loss of generality, we configure the reconfigurable element patches into columnar arrays where each column shares common positive/negative voltage rails (column-wise control). Due to the good isolation characteristics of the inductor, the influence of high impedance bias lines behind the inductor is not considered in the element design. The construction of the PC is shown in Figure 2b. A GF220 substrate ( $\varepsilon_{r}$= 2.2) with 2 mm thickness is selected. The bottom of the PC is a complete metal ground, and the top patch is slotted in the center with a slot width of 0.3 mm to solder diodes. In this design, the initial PC optimization objective is set as a 1-bit phase modulator in the Ku-band when FM Ⅰ is the air layer. Then the width of the patch is optimized to 3.35 mm, resulting in the final PC element.

As described in Figure 2g, different from traditional reconfigurable loads, it exhibits stable dispersion properties across the frequency domain. The PC generates strong dispersion with frequency variation and exhibits strong resonance points at specific frequencies. The electric field distribution of the PC in the ON and OFF states at 7 GHz, 10 GHz, 11.6 GHz, and 16.4 GHz is depicted in **Figure S1**. When the diode is biasing and turned off, the electric field is cut off by the diode, and a symmetrical electric field is induced at the edge of the slot. When the PC is in the ON state, the electric field exhibits continuity at both ends of the diode. Besides, it can be found that at the typical resonant frequency 11.6 GHz in the OFF state, shown in Figure S1e, due to the localized resonance of electromagnetic wave energy within subwavelength elements, a strong electric field distribution has formed on the patch. At these frequencies, the stored energy is released, and the impedance of the metasurface element exhibits a zero imaginary part characteristic. Similarly, when the PC is in the ON state, there are also instances of extremely high electric fields at 7 GHz and 16.4 GHz.

The physical images of the processed PC array are shown in Figure S2. Each column's bias is isolated from RF and DC by adding inductors at the end. 40 bias lines are led out through four 2 × 14 connectors at the bottom. After the PC is determined, the functional modules can be customized for the corresponding application.


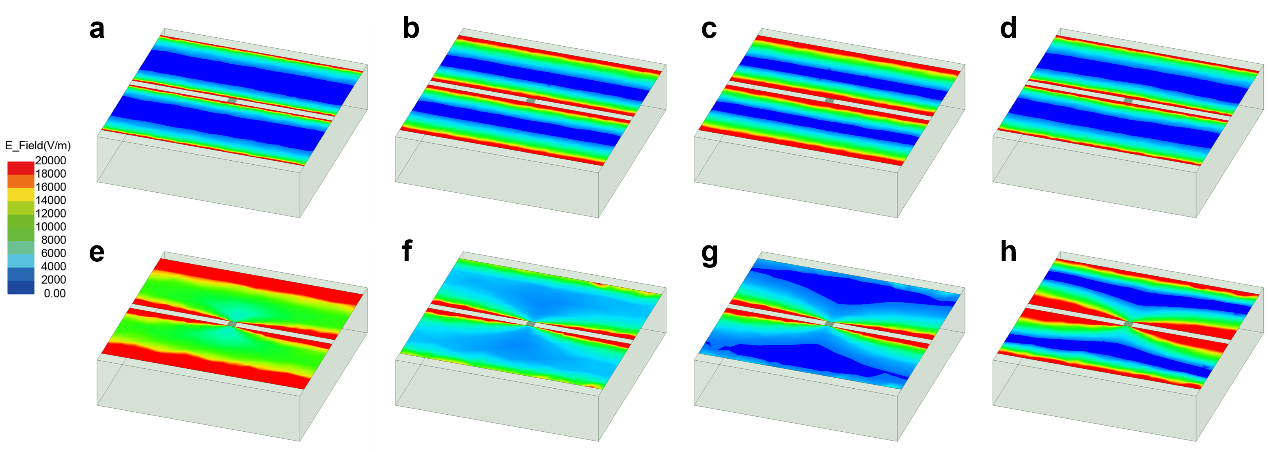


**Figure S1**. The electric field distribution of the PC and the fabrication of the prototype. The electric field distribution of the PC in the off state at a) 7 GHz, b) 10 GHz, c) 11.6 GHz, and d) 16.4 GHz. The electric field distribution of the PC in the on state at e) 7 GHz, f) 10 GHz, g) 11.6 GHz, and h) 16.4 GHz.


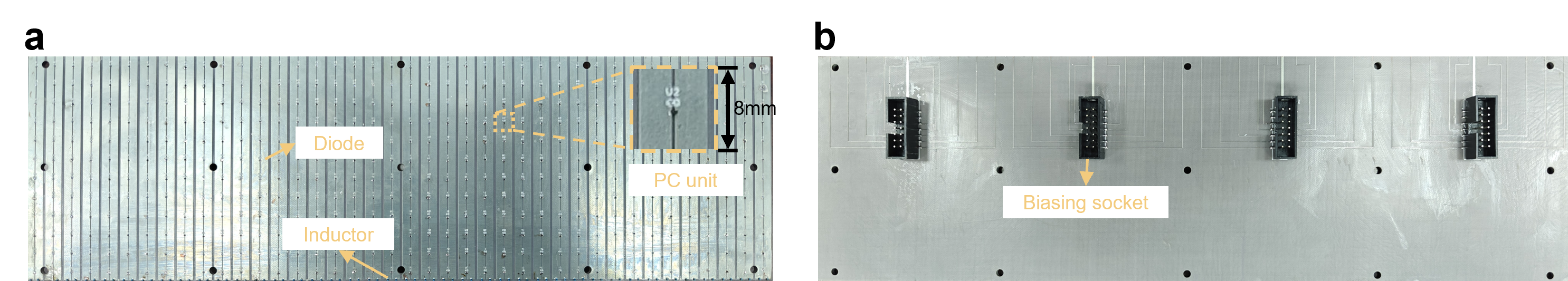


**Figure S2. The picture of the PC prototype.** a) The top view of prototype PC. b) The bottom view of prototype PC.

Supplementary Note 2. Fast analysis and design of metasurface elements based on HRMA

HRMA-based metasurface provides a method for rapid analysis and design of a reconfigurable metasurface element. PCs have two significant characteristics. Firstly, they can be flexibly reused as a transferable, reconfigurable load in any design. Secondly, different from the traditional switches, which change between short circuit and open circuit, PCs have the ability to switch between multiple states with specific impedance and a strong dispersion effect. This means that not only in array regulation, the encoding state of PC elements has the advantage of flexibility and agility, but in element design, multiple states of PCs can also be enumerated to obtain the load impedance under corresponding states.

In this design example, one PIN diode is applied in each PC, and it switches between two states, which are named on and off states as the diodes are turned on and off. Though the functional layer is changed according to the applications and scenarios, two load impedance states of the PC are attainable when the structure is determined as shown in Figure 2e. The total impedance of the metasurface can be obtained by cascading the specific functional layer and the PC. And as introduced in (3) and (4), the reflection coefficients can be calculated according to the total impedance.

Here, as an example, the cascade process of a medium ($\varepsilon_{r}$= 3.55) to the PC is depicted in the **Figure S3**. The medium is 3 mm thick and located 2 mm above the PC, as shown in the Figure S3a. The medium is simulated in PBC boundaries and assigned with Floquet port excitation at the top and bottom. It should be noted that the Floquet port at the bottom side should be de-embedded to include the required 2 mm air layer. The S-parameter results are shown in Figure S3b and c. With the network parameters extracted, the final impedance of the metasurface can be calculated by cascading the media and PCs. Figure S3d gives the cascade model in the on and off states, in which the impedance of PC is extracted at first and reusable in all cases. The cascade and simulation results of the whole structure, as shown in the Figure S3e and f, show good consistency, which supports the accuracy of this method and provides a foundation for subsequent independent, fast, and simplified simulations for the functional modules. In the design process, the simplified simulation of the functional layer can be conducted, and the results can be cascaded with the PC load. When the result is close to the design target, the final optimization and parameter fine-tuning of the functional metasurface can be carried out.


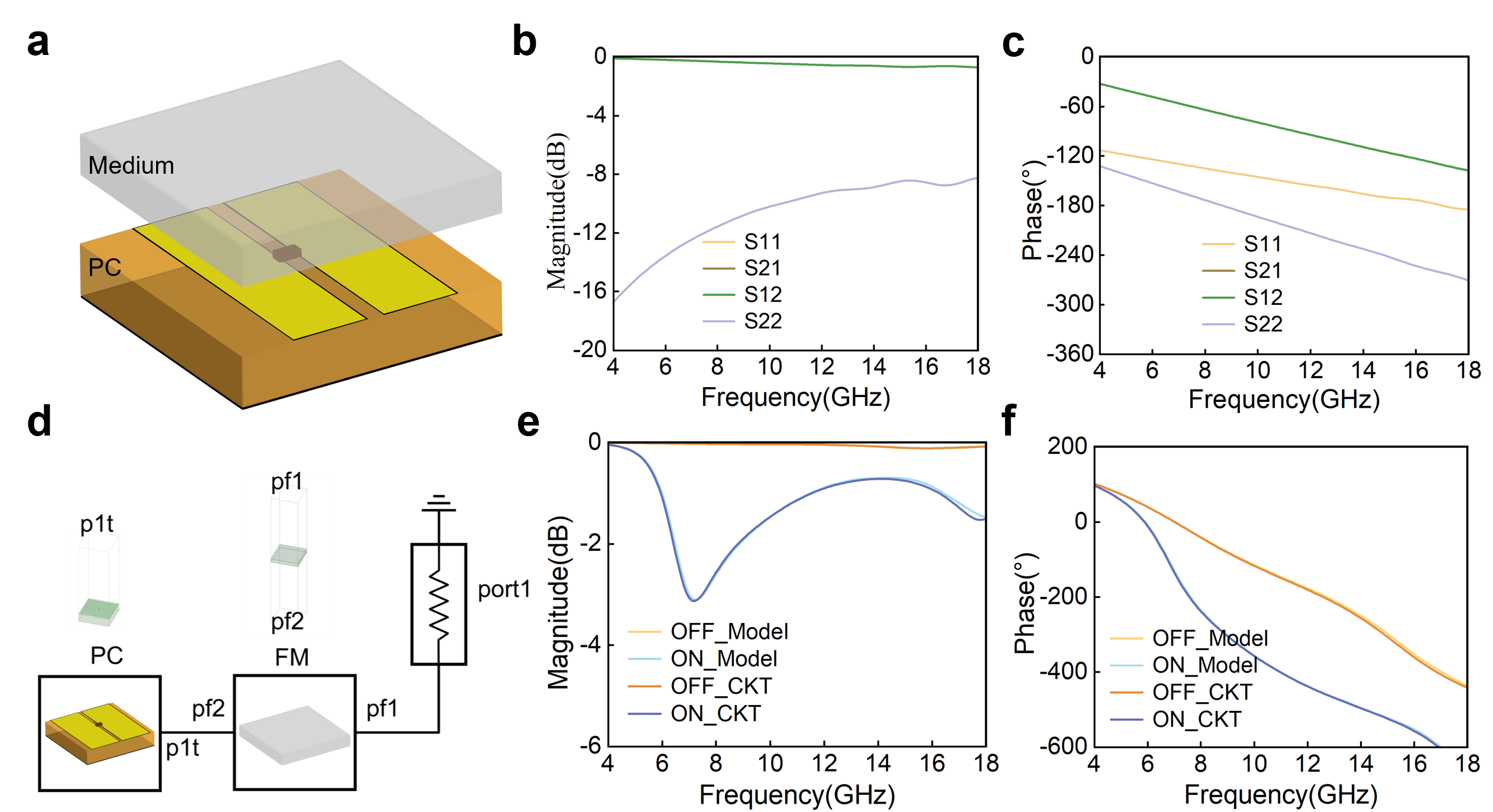


**Figure S3.** The verification of the fast analysis methods through cascading modules. a) The simulation model structure with a substrate loaded above the PC. The simulation b) magnitude, and c) phase of the medium. d) The circuit cascade model for simplifying and rapid analysis. e) and f), The simulated reflection coefficients of a) and d).

It can be seen that the functional layer and the reconfigurable layer are not only cascaded structurally, but also electromagnetically. Therefore, when designing the functional layer with a specified encoding layer, we only need to extract the network parameters and obtain the impedance of the PC at first. Then, the multiple impedance parameters obtained can be used as needed in subsequent designs. Through simulation optimization at the functional layer and cascading with load parameters, the design of any functional metasurface can be quickly achieved.

Supplementary Note 3. Design and configuration of FMs Ⅰ-Ⅲ for an ultra-wideband phase modulator

Although PC has arbitrariness as a reconfigurable load, in order to demonstrate more functionality and flexibility, the PC is first designed as a 1-bit phase modulator working at Ku-band, when the air layer is considered as FM Ⅰ. Further, functional modules are designed and appended to the structure to form the polymorphic function.

Firstly, the air layer is considered as functional layer Ⅰ, and 1-bit phase modulation is observed around 14 GHz in Figure 3d. According to different scenarios and application demands, the phase modulation function layer can be switched, migrating operation frequency domain.


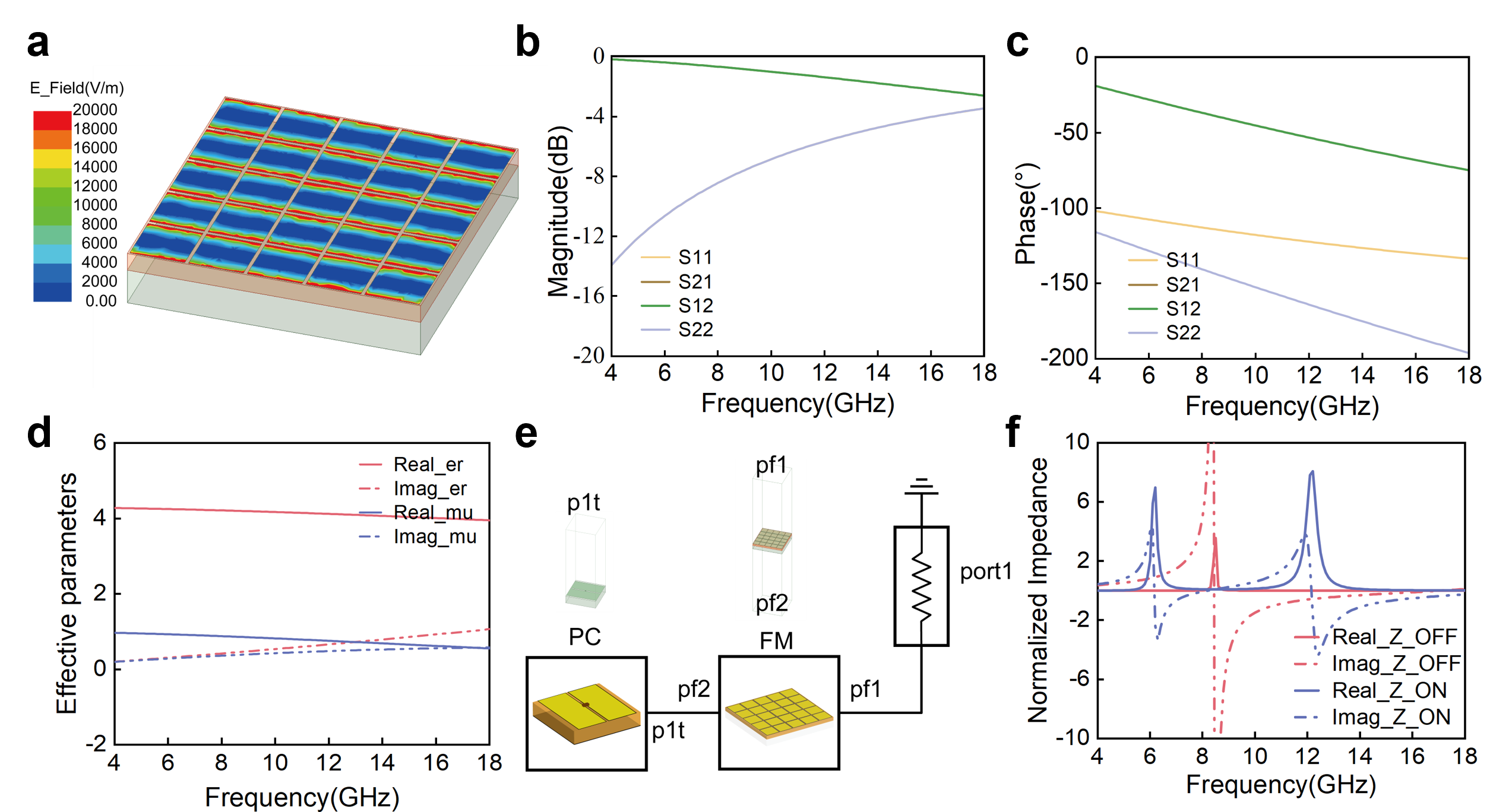


**Figure S4. The design process of FM Ⅱ.** a) The structure and the electric field of FM Ⅱ at 10 GHz. b) The cascade circuit model of the metasurface element for fast analysis. c) The simulated magnitude and d) phase of the layer. e) The final impedance of the metasurface element with FM Ⅱ.

FM Ⅱ is composed of periodic metal lattices, a dielectric layer ($\varepsilon_{r}$ = 2.2), and an air layer, as shown in Figure 3b. The width of the square patch is 1.5 mm, and the period of the lattices is 1.6 mm, less than λ/10 at the highest frequency. The height of the substrate and the air layer is 0.508 mm and 1 mm, respectively. The layer is simulated separately in the PBC boundaries. An electric field distribution in the form of a sine function on the surface is formed as depicted in the **Figure S4**a. The S-parameters deembedded to the top and bottom surfaces of the layer are obtained in the Figure S4b and c. Due to the absence of loss and ferromagnetic materials on the functional layer, it has both lossless and reciprocal characteristics, namely |S_12_|^2^+|S_21_|^2^=1, and S_12_=S_21_. Due to asymmetry, S_11_ and S_22_ have similar amplitudes, but there is a significant difference in phase. For macro-analysis, the equivalent parameters of this functional layer are extracted according to the KK-methods, which is

$$\varepsilon_{\text{eff}}=\frac{N_{\text{eff}}}{Z_{\text{eff}}},\mu_{\text{eff}}=N_{\text{eff}}Z_{\text{eff}}$$

$$N_{\text{eff}}=\frac{1}{k_{0}d_{\text{eff}}}\left\{\mathrm{Im}\left[\ln\left(e^{iN_{\text{eff}}k_{0}d_{\text{eff}}}\right)\right]+2m\pi-i\mathrm{Re}\left[\ln\left(e^{iN_{\text{eff}}k_{0}d_{\text{eff}}}\right)\right]\right\}$$

$$Z_{\text{eff}}=\pm\sqrt{\frac{\left(1+S_{11}\right)^{2}-S_{21}^{2}}{\left(1-S_{11}\right)^{2}-S_{21}^{2}}}$$

in which $k_{0}$ is the wave number in vacuum, and *d*_eff_ is the thickness of the medium. $\varepsilon_{\text{eff}}$ and $\mu_{\text{eff}}$ can be calculated by from the complex effective wave impedance $Z_{\text{eff}}$ and complex effective refractive index determined by and .

An effective medium of 1.508 mm length with a permittivity of 4.17+0.53i and permeability of 0.82+0.42i is achieved at 10 GHz. In fact, by designing and adding the FM Ⅱ, a transmission line is constructed, which introduces changes in the overall impedance of the metasurface. With the fast analysis method described in Note 2. The whole impedance of the metasurface can be calculated and depicted in the Figure S4e. And the normalized impedance is obtained as Figure S4f. The reconfigurable metasurface element undergoes distinct electromagnetic configurations during on and off state switching. Observing the total impedance, it can be found that at 8.5 GHz, the element in the off state exhibits resonant anomalies characterized by imaginary part oscillation and real part fluctuation, while the on-state manifests dual-frequency resonances at 5.4 GHz and 12.2 GHz. Equation (2) reveals that at resonance points (defined by Im(*Z*)=0 and ∂Re(*Z*)/∂*f*=0), the reflection coefficient magnitude approaches unity (|Γ|=1) with zero phase (θ=0°). In proximity to these resonances, a positive-to-negative phase transition occurs, where the reflection magnitude Γ becomes predominantly determined by the ratio of Re(Z) to the characteristic impedance $\eta_{0}$. Through such impedance design, the reflection coefficient magnitude and phase (Figure 2e) of the fundamental reconfigurable layer exhibit distinct operational characteristics: a 1-bit modulation bandwidth of 7–11.9 GHz with 180°±20° phase reversal accuracy, accompanied by reflection magnitude >-3 dB across the entire operational band.

Similarly, FM Ⅲ is designed, which consists of two substate layers ($\varepsilon_{r}$ = 3.55), two square lattices, and an air layer. The widths of the squares are 0.5 mm and 1.5 mm, and the heights of the substrates are 0.254 mm and 0.508 mm from top to bottom. The simulated results and design process are given in the **Figure S5**. For the experimental verification, FM Ⅱ and FM Ⅲ are processed with PCB technology, which is shown in the **Figure S6**.


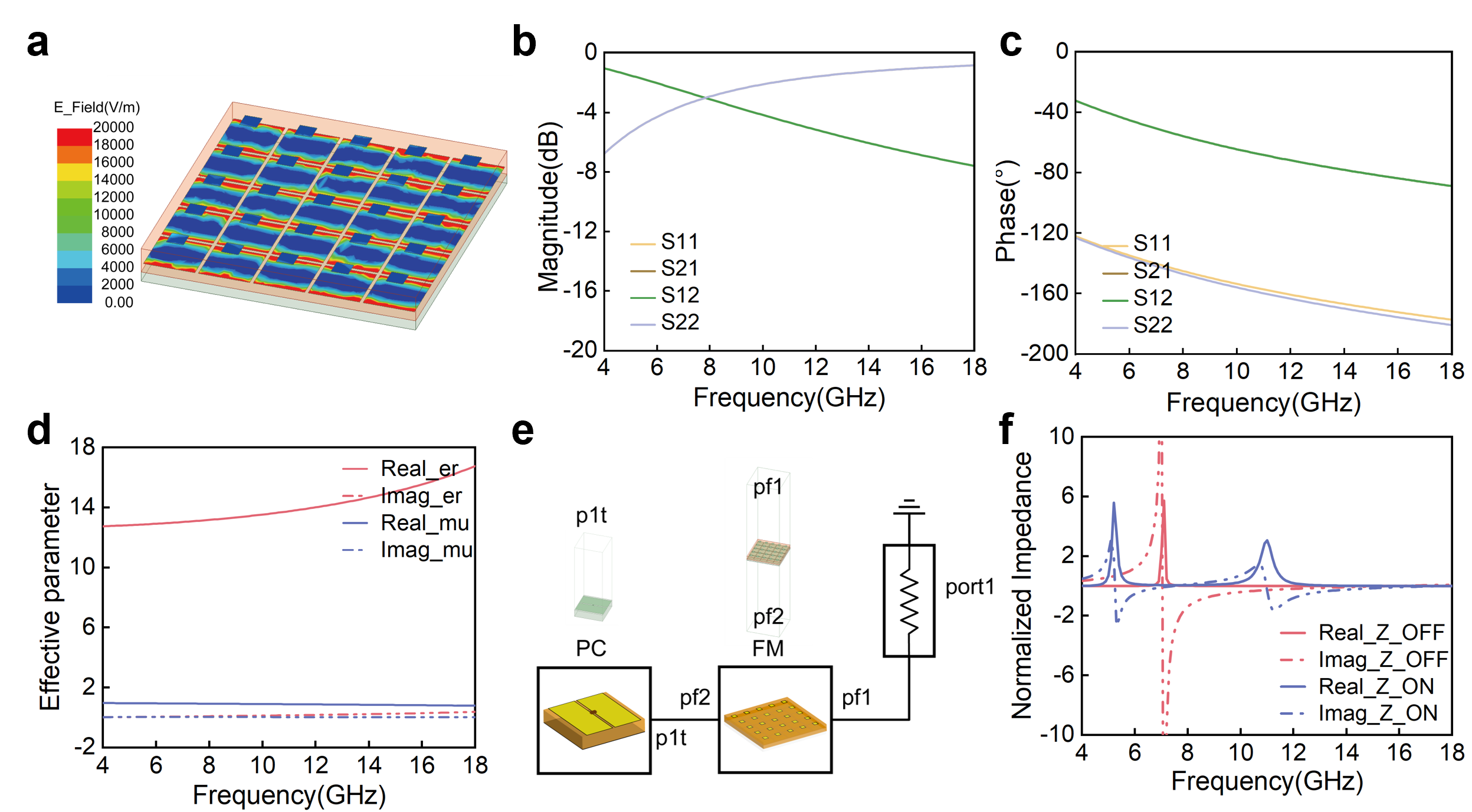


**Figure S5.** The design process of FM Ⅲ. a) The structure and the electric field of FM Ⅲ at 6 GHz. b) The cascade circuit model of the metasurface element for fast analysis. c) The simulated magnitude and d) phase of the layer. e) The final impedance of the metasurface element with FM Ⅲ.


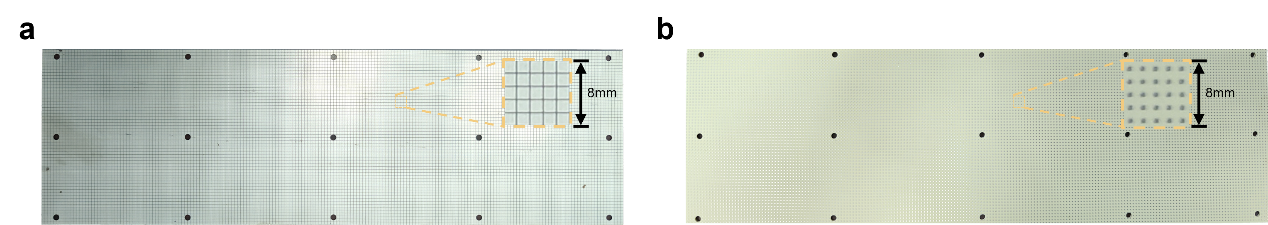


**Figure S6.** The picture of the FM Ⅱ and Ⅲ prototype. a) The top view of prototype FM Ⅱ. b) The top view of FM Ⅲ.

Supplementary Note 4. Beam modulation based on the space wave diffraction grating equation and generalized Snell’s law

When electromagnetic waves are incident on a periodic phase modulation surface, multiple spatial harmonics will be generated, and their scattering angles are constrained by the diffraction grating equation

$$\sin\theta_{m}=\sin\theta_{i}+\frac{2\pi m}{k_{0}p}=\sin\theta_{i}+\frac{m\lambda}{p}$$

In which *m* represents the *m*-th spatial harmonic, $\theta_{\text{r}}$ is the deflection angle of the harmonic, $\theta_{i}$ is the incident angle, and *k*_0_ is the wave number in the air. The phase distribution in one period is obtained using the generalized Snell's law.

$$\Phi_{r}=kx\left(\sin\theta_{i}-\sin\theta_{r}\right)+\Phi_{c}$$

In order to achieve the regulation of scattering beams within a broadband, an ultrawideband reconfigurable metasurface should be designed first. As introduced before, FM Ⅱ and Ⅲ expand the bandwidth of the metasurface, generating a working band from 5.6 GHz to 15.6 GHz with a relative bandwidth of 93%. Due to the dispersion effect, if the same encoding and modulation period are used, the angles of the harmonics generated will shift. To avoid this phenomenon, the phase distribution is calculated and quantified to 1-bit from 6-15 GHz at the desired frequencies. Here, a step of 1 GHz is selected, and the pattern is calculated based on , which is shown in Figure 3l.


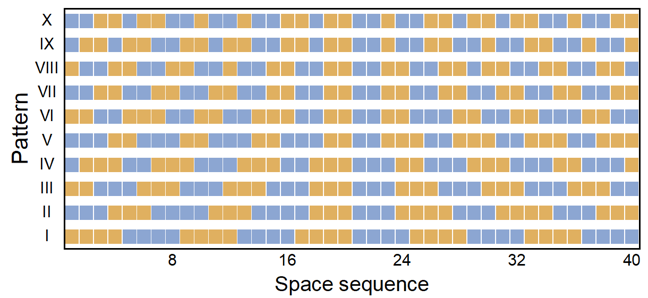


**Figure S7.** The corresponding pattern of the HRMS-based RMS, in which the pattern Ⅰ-Ⅹ corresponds to the integer frequency points of 6-15 GHz, respectively.

For comparison, a conventional metsurface element with 1-bit phase resolution working at 7 GHz is designed. **Figure S8a** shows the structure. The element is a typical 1-bit structure with a diode and resonant patch on the substrate, of which the permitivity is 2.2. The element period is 8 mm. The other design parameters are *h*_c_ = 2.5 mm, *w_x_* = 7.2 mm, *w_y_* = 7.4 mm. The reflection phase and amplitude at the ON and OFF states are shown in Figure S8b. An array of 40×12 scale is constructed, with the coding pattern calculated at 7 GHz.

**
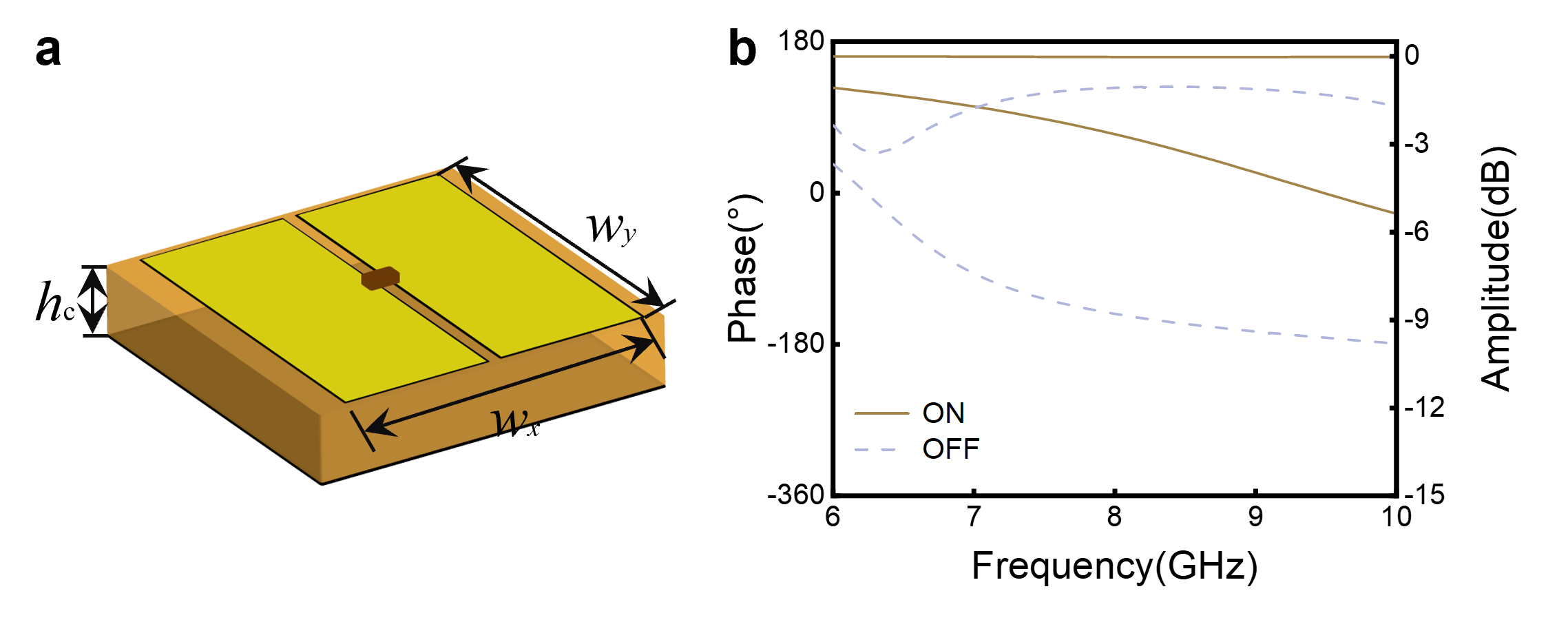
**

**Figure S8.** The conventional RMS element for comparison.(a) The element structure. (b) The reflection phase and amplitude.

Supplementary Note 5. Frequency modulation based on space-time-coding metasurface

The reconfigurable metasurface with 1-bit phase resolution has the capability of harmonic generation and frequency shift based on the spatiotemporal modulation strategy. According to space-time coding theory, the far-field pattern of the spatiotemporal metasurface with the reflection coefficient matrix $\Gamma_{pq}^{n}$ and the length of the time-coding sequence $L$ is

$$a_{qp}^{m}=\sum_{n=1}^{L} \frac{\Gamma_{pq}^{n}}{L}\sin c\left(\frac{\pi m}{L}\right)\exp\left[\frac{-j\pi m\left(2n-1\right)}{L}\right]$$

Here, to modulate the fundamental energy onto the -1^st^ harmonic and achieve an angle deviation of $\theta_{t}$, the compensation phase of the element and the delay time *t*_c_ is calculated by

in which *x* is the position of the element in the *x*-direction. Here, for the application demonstration, the target angle is set to -23°. The RMS with FM II is taken as the frequency modulator for a 10 GHz monochromatic wave incidence, with a modulation rate of 500 kHz. The final space-time coding matrix in this design is presented in Figure 3o.


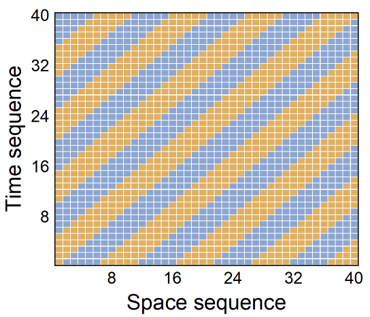


**Figure S9.** **The calculated space-time matrix to manipulate the -1^st^ harmonic to -23°.**

Supplementary Note 6. Design and configuration of FM Ⅳ for a broadband reconfigurable absorber

As shown in Figure 4a, consisting of a square resistive patch with a side length of 5 mm attached to a 1 mm thick GF220 dielectric($\varepsilon_{\text{r}}$ = 2.2), FM Ⅳ is located 4 mm above the PCs. According to (2), when the total impedance approaches 377 ohms, the reflection coefficient will approach its minimum value. To form a matching to the air wave impedance, a patch layer is sprayed with a 200 ohm/sq resistive film. The complex electric field distribution at 10 GHz is given in the **Figure S10**a. The simulated two-port S-parameters for FM IV are presented in the Figure S10b and c, from which the equivalent parameter characterization results of FM Ⅳ are obtained in Figure S10d. Obviously, through the loading of resistive materials, FM Ⅳ exhibits stable resistive behavior throughout the entire frequency range, with the real part of the equivalent permitivity remaining around 2 and the permeability remaining around 1. Figure S10e provides the simulation model of the metasurface design process. By cascading FM Ⅳ and the PC, the load impedance of the final element can be obtained, which is normalized and given in the Figure S10f and Figure S10g. Under TE incidences, from the total impedance shown in the Figure S10f, it can be learned that when the PC is turned on, there exist three resonant points, namely 5.3 GHz, 9.4 GHz, and 18 GHz, where the imaginary part of the impedance passes through 0 and the real part reaches a local maximum that is close to 1. The reflection coefficients exhibit local minima near these frequencies. When the PC is switched off, corresponding resonance points have also appeared, but these resonance points have shifted to around 5.8 and 15.4 GHz, which means that the operating frequency of the absorber will be adjusted. Similarly, the results when the element is excited by TM incidences are exhibited in the Figure S10g. At this time, resonance occurs in the X-band and remains in both states. Due to the reconfigurable property of the PC being *y*-polarization, when the PC switches states, almost no change can be observed in reflection performance. The top view of the prototype of FM Ⅳ is given in the Figure S10h.


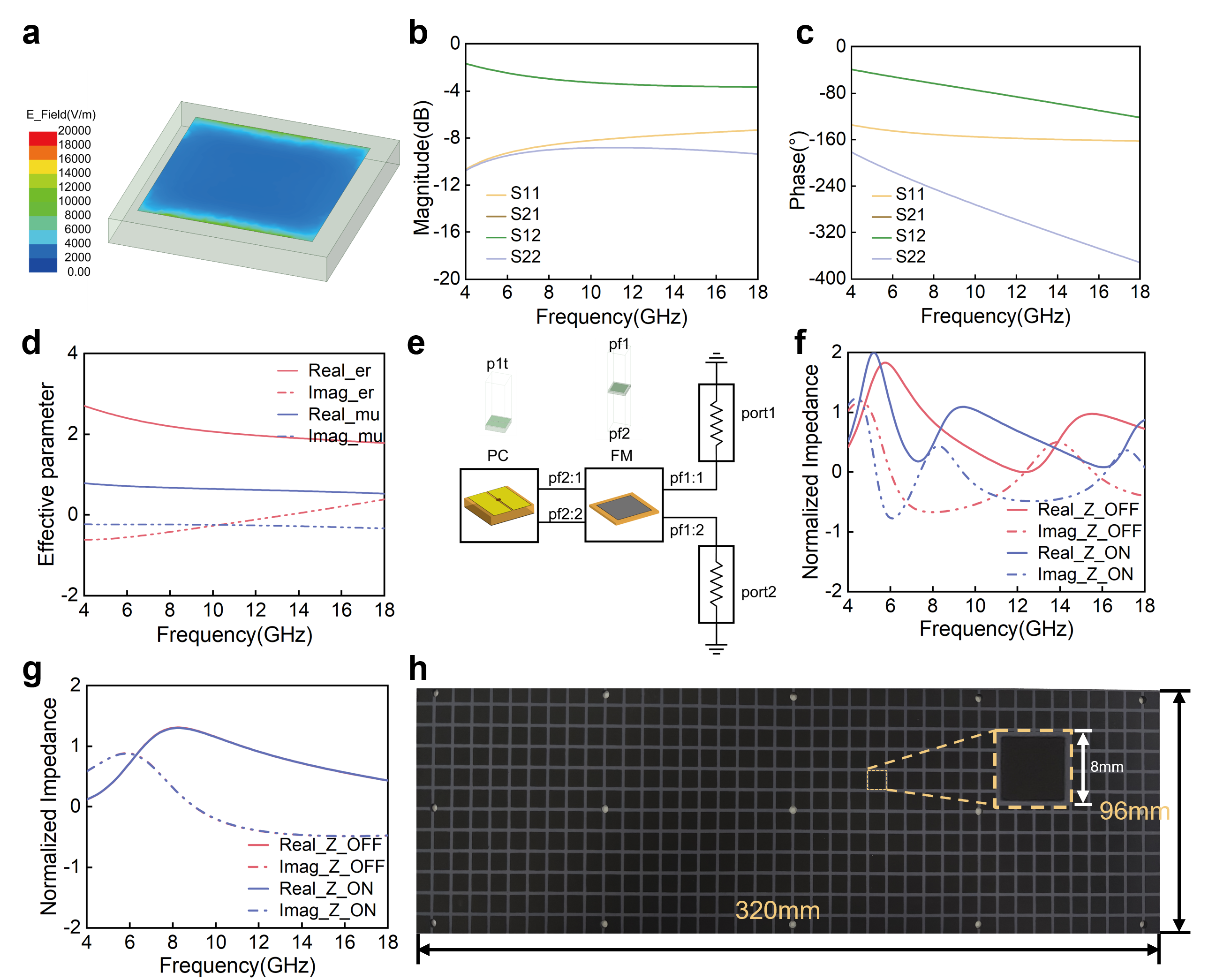


**Figure S10.** The design and configuration of FM Ⅳ. a) The structure and the electric field of the functional layer at 10 GHz. The simulated b) magnitude, and c) phase of S-parameters of FM Ⅳ. d) The cascade circuit model of the metasurface element for fast analysis. e) The effective constitutive parameters of the layer. The total impedance of the metasurface element with FM Ⅳ f) in *x*-polarized incidences. g) in ***y***-polarized incidences. h) The top view of FM Ⅳ prototype.

Supplementary Note 7. The synthesis of an adjustable absorber with the capability of tunable RCS modulation depth

With the reconfigurability of reflection coefficients of the element, the metasurface array constructed by FM IV and PC forms an adjustable absorber with flexibility. In this design, the absorption rate (AR) is calculated with

$$AR=1-\frac{\sigma_{DUT}}{\sigma_{REF}}$$

in which $\sigma_{DUT}$ is the RCS of the metasurface and $\sigma_{REF}$ is the RCS of the metal plate of equal size. The design of a broadband adjustable absorber is fundamentally formulated as a mathematical optimization problem for electromagnetic scattering control through systematic element proportion manipulation. The optimization framework treats the proportion parameters $\mathbf{p}=\{p_{1},p_{2}\}$ as decision variables subject to the physical constraint $p_{1}+p_{2}=1$ where$p_{1},p_{2}\in[0,1]$, enabling comprehensive control over the electromagnetic response through strategic distribution of on and off state elements. The primary objective functions encompass both RCS minimization, expressed as$\min_{\mathbf{p}}\sigma(f,\mathbf{p})=\frac{4\pi^{3}S^{2}}{\lambda^{2}}|p_{1}r_{1}(f)+p_{2}r_{2}(f)|^{2}$, and absorption maximization, formulated as$\max_{\mathbf{p}}A(f,\mathbf{p})=1-|p_{1}r_{1}(f)+p_{2}r_{2}(f)|^{2}$, where $r_{1}(f)$ and $r_{2}(f)$ represent the frequency-dependent complex reflection coefficients of the two element types.

$$\mathbf{p}=\{p_{1},p_{2}\} \text{where }p_{1},p_{2}\in[0,1], p_{1}+p_{2}=1$$

$$\min_{\mathbf{p}} \sigma(f,\mathbf{p})=\frac{4\pi^{3}S^{2}}{\lambda^{2}}|p_{1}r_{1}(f)+p_{2}r_{2}(f)|^{2}$$

$$\max_{\mathbf{p}} A(f,\mathbf{p})=1-|p_{1}r_{1}(f)+p_{2}r_{2}(f)|^{2}$$

The theoretical foundation underlying this optimization approach derives from coherent scattering superposition principles, where the total electromagnetic response is given by$E_{s}^{total}=\frac{jk_{0}N}{4\pi R}\exp(-jk_{0}R)(p_{1}r_{1}+p_{2}r_{2})$, enabling direct mathematical correlation between proportion parameters and observable electromagnetic characteristics. For perfect absorption at any target frequency$f_{i}$, the optimal condition requires$p_{1}r_{1}(f_{i})+p_{2}r_{2}(f_{i})=0$, which can be analytically solved to yield the optimal proportion configuration as $p_{1}^{*}(f_{i})=|r_{2}(f_{i})|/(|r_{1}(f_{i})|+|r_{2}(f_{i})|)$ and $p_{2}^{*}(f_{i})=|r_{1}(f_{i})|/(|r_{1}(f_{i})|+|r_{2}(f_{i})|)$

$$E_{s}^{total}=\frac{jk_{0}N}{4\pi R}\exp(-jk_{0}R)(p_{1}r_{1}+p_{2}r_{2})$$

$$p_{1}r_{1}(f_{i})+p_{2}r_{2}(f_{i})=0$$

$$p_{1}^{*}(f_{i})=\frac{|r_{2}(f_{i})|}{|r_{1}(f_{i})|+|r_{2}(f_{i})|}, p_{2}^{*}(f_{i})=\frac{|r_{1}(f_{i})|}{|r_{1}(f_{i})|+|r_{2}(f_{i})|}$$

To achieve broadband performance across the frequency set, the optimization strategy extends to multi-frequency objectives expressed as, where $w_{i}$ represent frequency-specific weights enabling prioritized optimization across different spectral regions. The experimental validation demonstrates exceptional performance with 11 distinct proportion patterns achieving absorption rates exceeding 0.7 across the entire 4-18 GHz frequency range, while frequency-selective optimization enables adaptive stealth performance tailored to different radar threat scenarios through real-time proportion adjustment capabilities.

Supplementary Note 8. Design and configuration of FM Ⅴ for a reconfigurable polarization converter

FM Ⅴ transforms the metasurface into a polarization converter. As shown in Figure 5a, a 45°-oriented rectangle patch is attached to a 0.508 mm-thick GF220 substrate above a 2.5 mm-thick air layer, forming FM Ⅴ. The FM Ⅴ is simulated in PBC boundaries, with TE and TM modes assigned from the top and bottom. The electric field distribution at TM incidences is shown in the **Figure S11**a. Under TM mode incidence, the electric field of the cross-polarization component is significant, causing a substantial enhancement of the polarization conversion component. Figure S11b and c provide the magnitude and phase results of the S-parameter when a TM mode wave is incident. As mentioned above, FM Ⅴ causes a considerable polarization conversion, which cannot be ignored. An extraction method of bianisotropic metamaterials can be applied, from which the constitutive parameter tensor of FM Ⅴ can be obtained. For ease of analysis, Figure S11e and S11f extract the material properties of the ±45° polarization components, owing to low polarization conversion coefficient. Finally, the transfer impedance of the metasurface is obtained as shown in the Figure S11g. As there is a relationship between polarization conversion parameters and transfer impedance, as follows

$$S_{21}=\frac{2Z_{21}Z_{0}}{\left(Z_{11}+Z_{0}\right)\left(Z_{22}+Z_{0}\right)-Z_{12}Z_{21}}$$

Therefore, when PC is in the off state, *S*_21_ approaches 0 when *Z*_21_ is minimum at 13.8 GHz, while in the on state, a broadband polarization transition characteristic of 10-14 GHz appears. Fabricated with PCB technologies, the FM Ⅴ prototype is depicted in the Figure S11h.


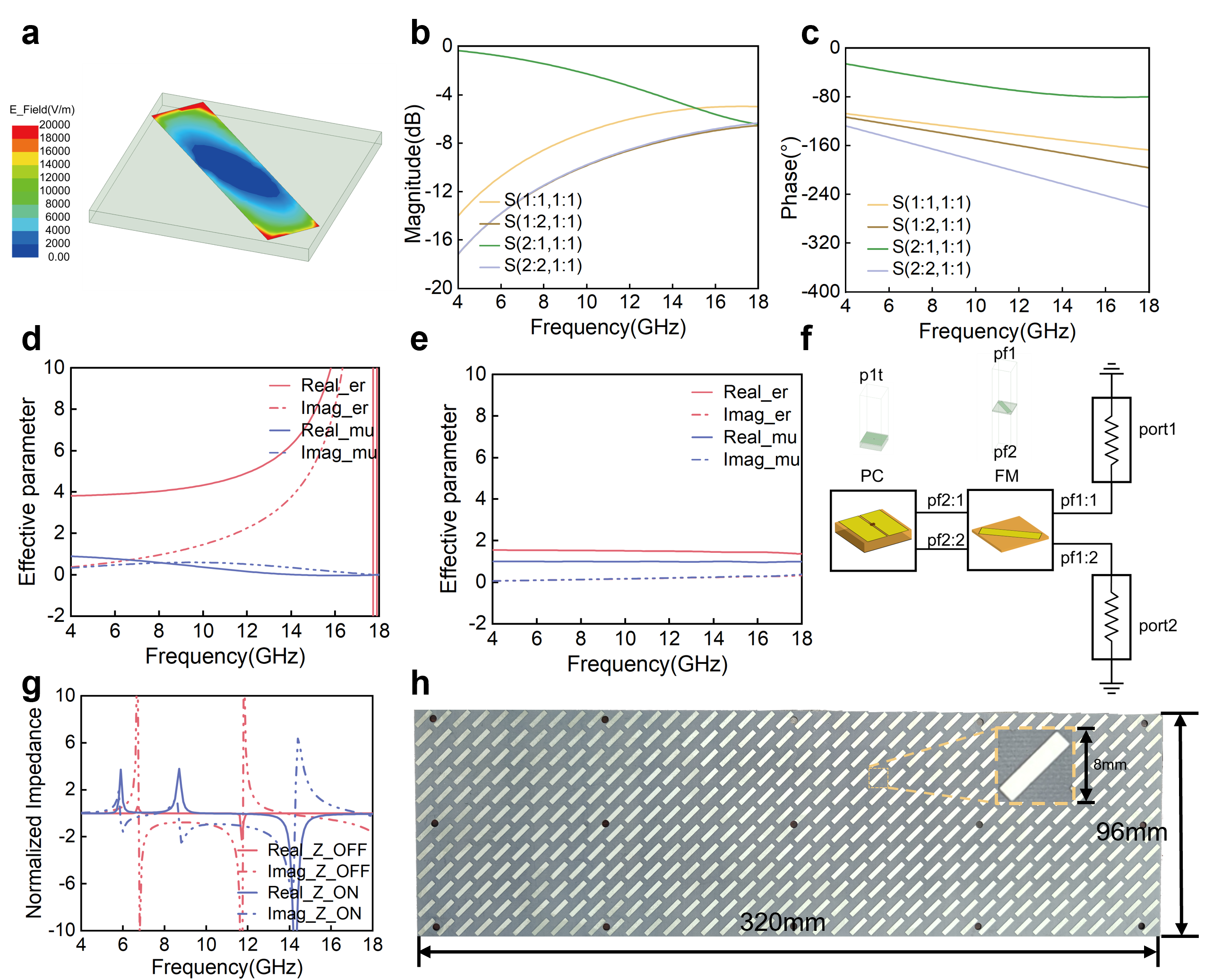


**Figure S11.** The design and configuration of FM Ⅴ. a) The structure and the electric field of the functional layer Ⅴ at 14 GHz under normal TM incidences. The simulated b) magnitude, and c) phase of S-parameters of FM Ⅴ. d) The cascade circuit model of the metasurface element for fast analysis. e) The effective permittivity, and f) the permeability of the layer. g) The total transfer impedance of the metasurface element with FM Ⅴ. h) The top view of FM Ⅴ prototype.

Supplementary Note 9. The synthesis and realization of a linear polarization converter with any polarization angle

The metasurface shows a polarization conversion and non-conversion ability at the element level. And verification is carried out on a 12×40 array. By measuring the RCS level of the prototype, we obtain the scattering field level $r_{cross}$ and $r_{co}$, further calculate the polarization conversion efficiency and polarization angle of the scattered field.

$$\eta=\frac{|r_{cross}|^{2}}{|r_{cross}|^{2}+|r_{co}|^{2}}$$

Experimental validation employs 11 distinct proportion patterns in a finite array at 13.8 GHz with horizontally polarized incidence, demonstrating maximum polarization conversion efficiency $\eta>90\%$ across 8.9-14.7 GHz and measured rotation angles . When the prototype array is large enough, the performance is expected to possess polarization conversion capabilities similar to those of elements in an infinite periodic array. Besides, according to symmetry, when FM Ⅴ is oriented to -45°, the polarization conversion will still be achieved in the on state and keep a 180° phase difference with the situation that FM Ⅴ is oriented to +45°, as shown in **Figure S12**a. On this basis, any linear polarization component in the *xoy* plane can be synthesized from these three polarization basis components. The simulation results of the mirrored element are provided in the Figure S12c and S12d. This provides a solid foundation for the realization of a polarization conversion in any polarization direction.


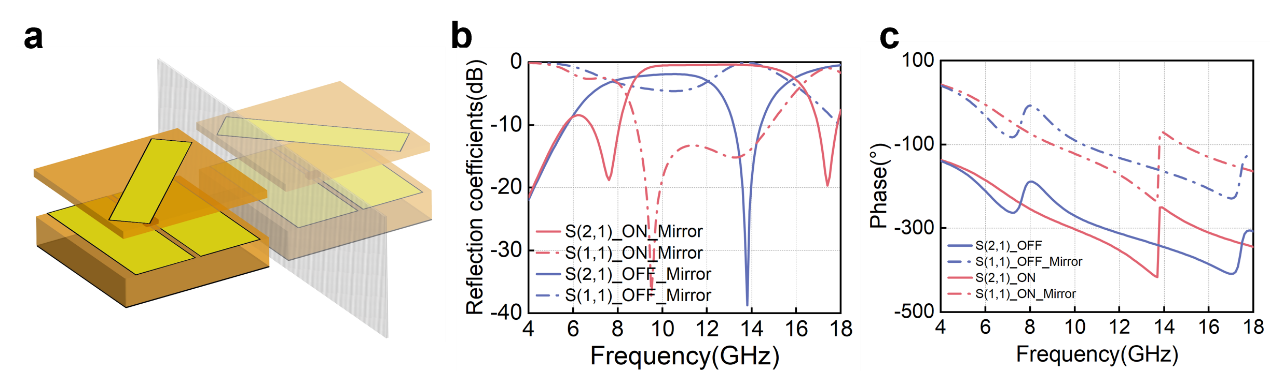


**Supplementary Figure S12.** The properties when FM Ⅴ is mirrored. a) The mirrored structure. b) Reflection coefficients of the metasurface when FM Ⅴ is mirrored. c) The phase difference of the polarization conversion coefficient when FM Ⅴ is mirrored before and after.

Based on the ±45° element, the verification of polarization conversion capability with arbitrary polarization angles is formulated as an angular targeting optimization problem for systematic element proportion control in the array. The mathematical framework treats the proportion parameters $\mathbf{p}=\{p_{1},p_{2}\}$ as decision variables subject to $p_{1}+p_{2}=1$ and$p_{1},p_{2}\in[0,1]$, targeting the achievement of any desired polarization angle through minimization of the objective function.

$$\mathbf{p}=\{p_{1},p_{2}\} \text{subject to }p_{1}+p_{2}=1, p_{1},p_{2}\in[0,1]$$

The system model characterizes the electromagnetic response through a reflection coefficient matrix $\mathbf{R}(\mathbf{p})=p_{1}\mathbf{R}_{1}+p_{2}\mathbf{R}_{2}$ for mixed element distributions, where $\mathbf{R}_{1}$ and $\mathbf{R}_{2}$ represent the reflection matrices of on and off state elements respectively. The cross-polarization component is expressed as$r_{cross}(\mathbf{p})=p_{1}r_{1,xy}+p_{2}r_{2,xy}$, while the polarization conversion efficiency follows$\eta(\mathbf{p})=|r_{cross}(\mathbf{p})|^{2}/(|r_{cross}(\mathbf{p})|^{2}+|r_{co}(\mathbf{p})|^{2})$, and the achievable polarization rotation angle is calculated as.

$$\mathbf{R}(\mathbf{p})=p_{1}\mathbf{R}_{1}+p_{2}\mathbf{R}_{2}=\left(\begin{matrix} r_{xx} & r_{xy} \\ r_{yx} & r_{yy} \end{matrix}\right)$$

$$r_{cross}(\mathbf{p})=p_{1}r_{1,xy}+p_{2}r_{2,xy}$$

$$\eta(\mathbf{p})=\frac{|r_{cross}(\mathbf{p})|^{2}}{|r_{cross}(\mathbf{p})|^{2}+|r_{co}(\mathbf{p})|^{2}}$$

The theorem establishes that for any target angle, there exists an optimal proportion configuration $\mathbf{p}^{*}$ satisfying, proven through the continuous mapping which ensures coverage completeness via continuity of reflection coefficient matrix elements, monotonic relationships between proportion parameters and achievable angles, and well-defined boundary conditions. The scalability analysis reveals that As the metasurface scale $N\to\infty$, the proportion control resolution approaches continuous values with $\Delta p_{min}=1/N\to0$, enabling enhanced angular targeting precision and theoretically complete first quadrant coverage expressed as. This establishes the theoretical foundation for comprehensive polarization control through proportion optimization.

**Supplementary Note 10. Analysis of the impact of assembly errors on the performance of HRMA-based RMS**

In this design, the PC here is separated from FM by air layer spacing. In fact, physical deviation and damage of element may occur between PC and FM after multiple assembly processes.


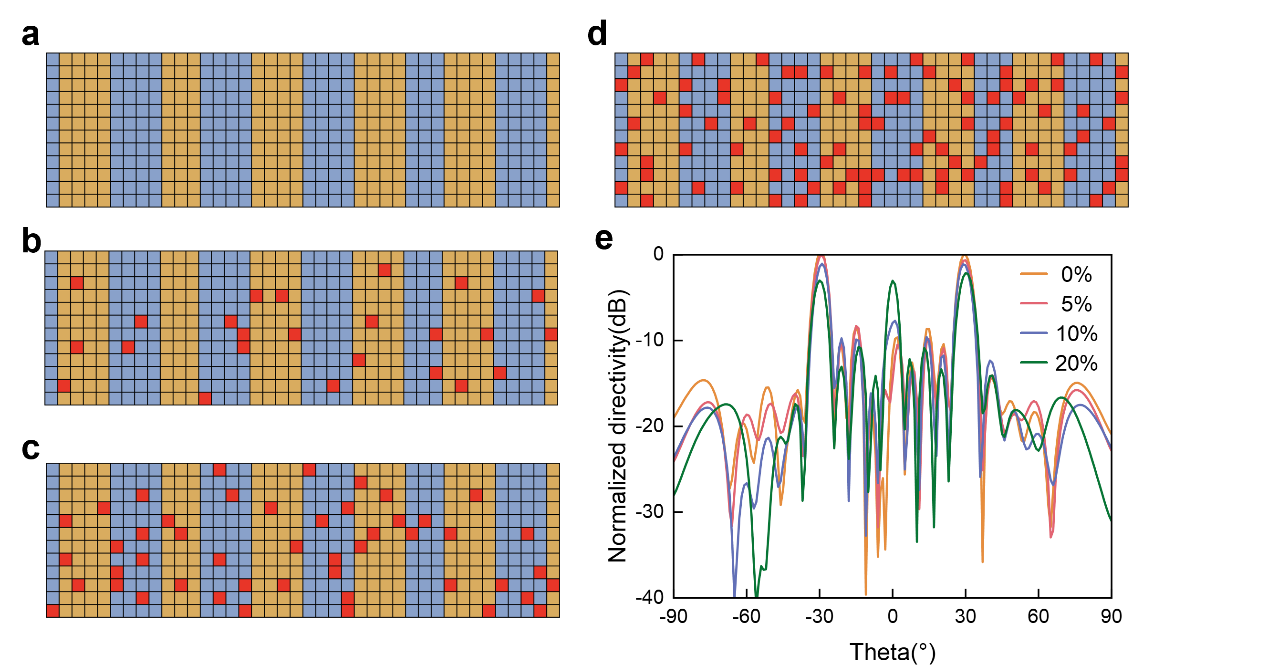


**Figure S13.** The simulation of RMS consisting of PC and FM II with different damage rate. (a) The coding pattern. (b)-(d) The coding pattern with 5%, 10% and 20% random damage elements. (e) The simulated results.

PCs are the most important components in the scheme, which may be damaged during multiple FM replacement. In order to illustrate the possible problems, PCs loaded with FM II is taken as an example for analysis. In the application scenario, possible damage rates of 5%, 10%, and 20% with random distribution are in Figure S13a-d. The simulation results is shown in Figure S13e. It can be seen that, when the damage rates is smaller than 5%, there is almost no difference in the main lobe and side-lobe compared to the expected results. When the rates increase to 10% and 20%, performances will deteriorate, such as the side-lobe level rising and the main lobe level falling, but the overall trend can still be maintained. As a result, a small number of diode damage before and after assembly will not significantly affect the performance.

Also, the lifetime of switching diodes should be considered, which mainly depends on their type, operating conditions (such as temperature, current and frequency), packaging technology and application scenarios. The service life varies greatly among different types of switching diodes. However, they can achieve a service life of more than 4 years in most scenarios, which indicates that the PC will have a similar lifetime under undisturbed conditions. However, diodes may fail during rough disassembly and installation of the FM, including solder detachment and direct diode damage. As previously noted, when the number of damaged diodes is small—i.e., less than 10%—the overall performance of the array degrades slightly. If a greater number of diodes are damaged, repair should be considered. In the case of solder detachment, re-soldering can be performed directly. Generally, the soldering cost is about ¥2 per diode. In contrast, direct diode damage, such as electrical failure or fracture caused by rough impact, is extremely rare. If such a condition occurs, the diode must be replaced with a new one and re-soldered.

Besides, the alignment error should be also considered. Here, the relative offset of FM is analyzed with PC as the coordinate center. According to industry standards, the hole diameter error of PCB drilling is within ±0.1 mm, and the air layer used in this work is formed with nylon nuts at intervals, and its thickness tolerance is within ±0.2 mm. As a result, certain performance variations will happen. The RMSs consisting of PC, FM II, and FM VI are chosen to illustrate the variation.

Figure S14a and d show the possible vertical and horizontal alignment errors when the PC is covered with FM II and VI. Figure S14b and c show the variation when FM II is loaded. The two kinds of alignment errors do not cause the characteristics of the curve to change, but move with the frequency, and the change caused by vertical error is slightly more obvious. A maximum phase variation of 11° is observed when the vertical alignment error is 0.2mm at 11.2 GHz. And for the FM VI loaded situation, the same trend is observed as shown in Figure S14e and f. The absorption bandwidth and absorption value remain basically unchanged, but the whole reflection coefficients have a frequency shift within the frequency band, reaching about 0.4 GHz at the maximum when the vertical errors reach 0.2 mm.

As a result, the assembly errors should be considered in HRMA during design and measurement. In order to ensure low vertical and horizontal errors, air foam, more fixed holes, or processes with higher accuracy can be used for FM installation.


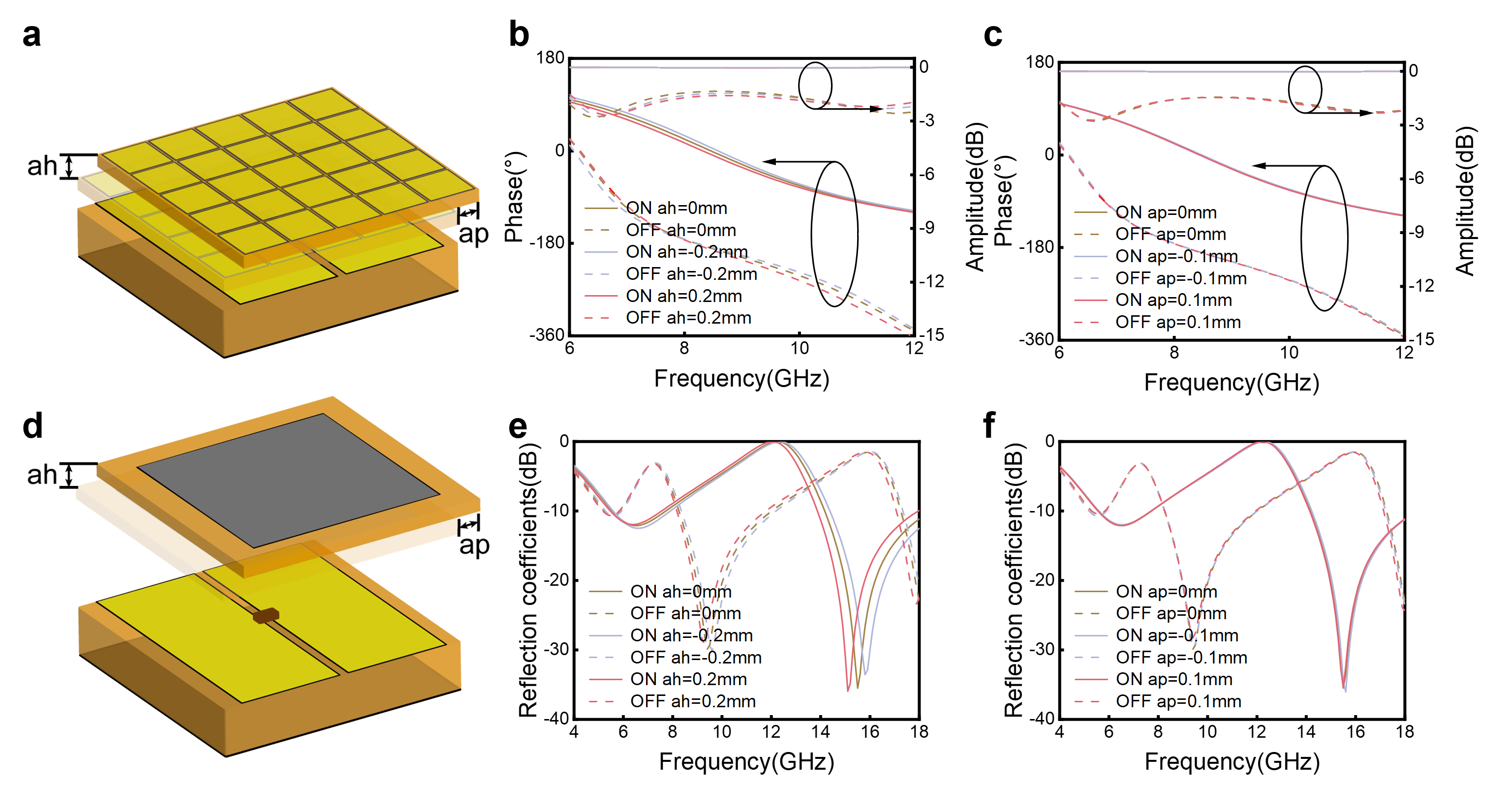


**Supplementary Figure S14.** The performance variation caused by the possible assembly errors. (a) The schematic of vertical and horizontal alignment when FM I is loaded. The phase variation under (b) vertical alignment error, and (c) horizontal alignment error. (d) The schematic of vertical and horizontal alignment when FM VI is loaded. The phase variation under (e) vertical alignment error, and (f) horizontal alignment error.
